# TDP1-independent pathways in the process and repair of TOP1-induced DNA damage

**DOI:** 10.1038/s41467-022-31801-7

**Published:** 2022-07-22

**Authors:** Huimin Zhang, Yun Xiong, Dan Su, Chao Wang, Mrinal Srivastava, Mengfan Tang, Xu Feng, Min Huang, Zhen Chen, Junjie Chen

**Affiliations:** Department of Experimental Radiation Oncology, The University of Texas MD Anderson Cancer Center, Houston, TX 77030 USA

**Keywords:** DNA damage response, Double-strand DNA breaks

## Abstract

Anticancer drugs, such as camptothecin (CPT), trap topoisomerase I (TOP1) on DNA and form TOP1 cleavage complexes (TOP1cc). Alternative repair pathways have been suggested in the repair of TOP1cc. However, how these pathways work with TDP1, a key repair enzyme that specifically hydrolyze the covalent bond between TOP1 catalytic tyrosine and the 3’-end of DNA and contribute to the repair of TOP1cc is poorly understood. Here, using unbiased whole-genome CRISPR screens and generation of co-deficient cells with TDP1 and other genes, we demonstrate that MUS81 is an important factor that mediates the generation of excess double-strand breaks (DSBs) in TDP1 KO cells. APEX1/2 are synthetic lethal with TDP1. However, deficiency of APEX1/2 does not reduce DSB formation in TDP1 KO cells. Together, our data suggest that TOP1cc can be either resolved directly by TDP1 or be converted into DSBs and repaired further by the Homologous Recombination (HR) pathway.

## Abstract (web summary)

Here the authors find that MUS81 mediates excess DNA double strand break (DSB) generation in TDP1 KO cells after camptothecin treatment. They show that TOP1 cleavage complexes can be either resolved directly by TDP1 or be converted into DSBs and repaired further by the Homologous Recombination pathway.

## Introduction

Genetic information is stored in double-helix–structured DNA molecules. When DNA replication, transcription, or recombination occurs, unwinding of double-stranded DNA is required, during which topological stresses are generated. DNA topoisomerases are types of enzymes that can specifically resolve topological stresses by transiently introducing strand breaks into DNA molecules and enabling the rotation of the supercoiled DNA strand^1,2^. Mammalian cells encode two types of topoisomerases: type I topoisomerases (TOP1, TOP1mt, TOP3α, and TOP3β), which introduce single-strand breaks into DNA, and type II topoisomerases (TOP2α, TOP2β, and SPO11), which introduce double-strand breaks (DSBs) into DNA^1–3^. During cleavage reaction, the tyrosine in the catalytic active site of topoisomerases is covalently linked to the DNA backbone and forms the so-called topoisomerase cleavage complex (TOPcc)^1,4^. A wide variety of topoisomerase poisons have been developed and have been used as chemotherapeutic drugs in cancer treatment. Many of them act to stabilize TOPcc, and lead to DNA strand breaks and eventually kill tumor cells.

TOP1 is highly conserved from prokaryotes to eukaryotes. It is ubiquitously expressed and is essential in mammalian cells^5^. The critical function of TOP1 is to relieve both positive and negative DNA supercoiling generated during transcription and replication and possibly during DNA repair and chromatin remodeling^1,5^. Camptothecin (CPT) is a natural product that can trap the TOP1 cleavage complex, and its derivatives have been approved for the treatment of ovarian, lung, and colorectal cancers^6,7^. Trapped TOP1cc blocks DNA transaction and can be converted into DNA breaks when it collides with a replication fork or transcription complex. Many repair factors may be involved in the repair of TOP1-induced damage, whose deficiencies cause cellular sensitivity to TOP1 poisons^4^. Therefore, further identification of key genetic factors that participate in the cellular response to TOP1-induced damage and characterize the relationship among these repair factors should provide a rational and optimal application of TOP1 poisons in cancer therapy.

TOP1-induced damage can be considered a type of enzymatic DNA-protein crosslink (DPC)^8^. The covalently bound TOP1 protein can be hydrolyzed by proteasome or proteases, such as Wss1 and SPRTN (SprT-like domain at the N-terminus). Tyrosyl-DNA phosphodiesterase 1 (TDP1) specifically resolves the covalent bond between TOP1 catalytic tyrosine and the 3’ end of DNA and therefore plays a unique and specific role in the repair of TOP1cc^9–11^. TDP1 is ubiquitously expressed and highly conserved in eukaryotes. TDP1 was the first discovered “precision scissor” that is capable of removing TOP1cc and thereby precisely liberating the stalled topoisomerase from DNA termini without cleaving DNA^11^. The removal of TOP1cc by TDP1 leaves a 3’-phosphate, which prevents TDP1 from removing another nucleotide^11^. TDP1 mutation was reported to be responsible for spinocerebellar ataxia with axonal neuropathy, a rare neurodegenerative disease^12^, suggesting that TDP1 has a physiological role in specific tissues. However, TDP1 deficiency is overall well-tolerated in vertebrates, since TDP1-deficient vertebrate cells show normal cell growth and knockout mice are viable, with no obvious phenotypes^13^. Thus, targeting TDP1 is expected to result in minimal toxicity but has the potential to selectively sensitize cancer cells over normal cells to TOP1 poisons^14,15^. As TDP1-deficient cells show hypersensitivity to topoisomerase poisons, it is clinically appealing to combine well-established topoisomerase poisons with TDP1 inhibitor for cancer therapy^14,15^. Thus, a lot of effort has been devoted to developing TDP1 inhibitors^14,15^.

Besides TDP1, the endonucleases involved in the nucleotide excision repair and homologous recombination (HR) pathways have also been demonstrated to excise TOP1cc^4,16,17^. However, it remains poorly understood how these pathways work with TDP1 and contribute to the repair of TOP1cc.

In this study, we investigated the mechanisms underlying the repair of TOP1-induced DNA damage, with or without TDP1. Interestingly, we detected increased DSBs in TDP1-KO compared with those in control wild-type cells, which were associated with the hyper-activated DSB-induced DNA damage response (DDR). Next, using unbiased whole genome CRISPR screens and the generation of co-deficient cells with TDP1 and other genes, we demonstrated that MUS81 is an important factor that mediates the generation of excess DSBs in TDP1-KO cells. These excess DSBs are further processed by several nucleases, such as MRE11, CtIP, DNA2, XPF, SLX4, or MUS81, to initiate HR repair. Thus, our study uncovers a conversion of accumulated TOP1cc into DSBs in the absence of TDP1, which promotes the activation of HR pathway for the repair of TOP1-associated DNA damage. These results may explain the mild sensitivity of TDP1 loss in response to TOP1 poison in proliferating cells. In addition, we showed that co-inhibition of TDP1 and DSB repair pathways enhanced cellular sensitivity to CPT treatment, which provides guidance for the further development of TDP1 inhibitors for cancer therapy.

## Results

### TDP1-KO cells show increased DSB formation and DSB-induced DNA damage response after treatment with TOP1 poison

**Fig. 1:**
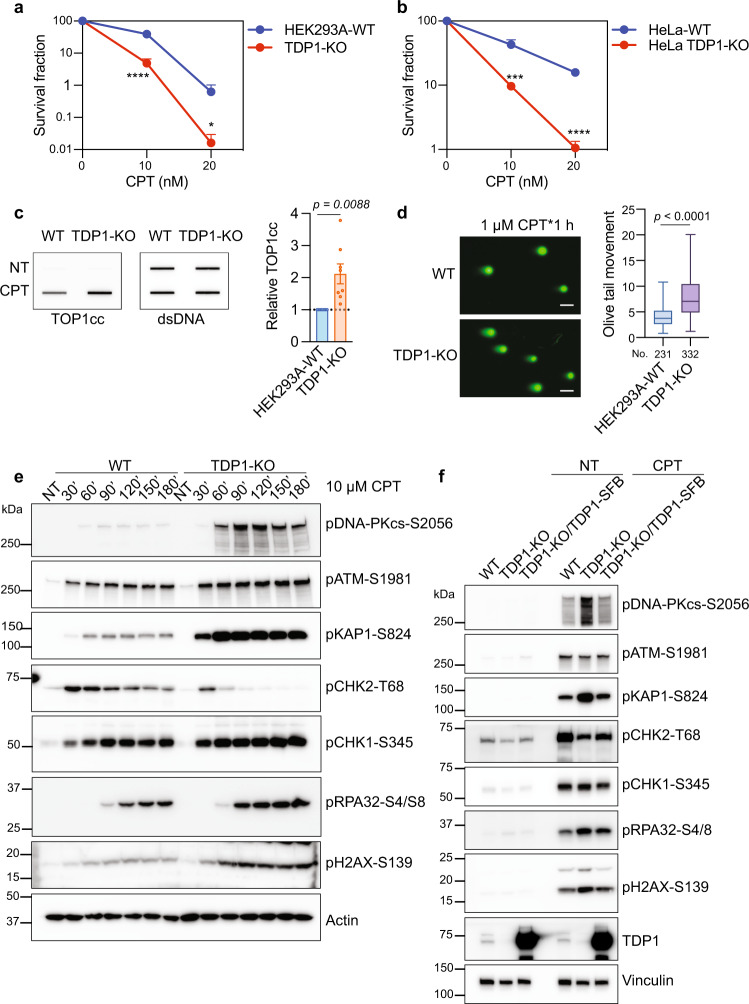
TDP1-KO cells show increased DSB formation and DSB-induced DNA damage response after treatment with TOP1 poison. **a** Colony formation assay of HEK293A-WT and TDP1-KO cells with CPT treatment. Data are represented as mean ± SD. *n* = 6 biologically independent replicates. Two-tailed unpaired *t* test with Welch’s correction was used for statistical analysis. ****p (10 nM CPT) = 0.000003 and *p (20 nM CPT) = 0.013413. **b** Colony formation assay of HeLa-WT and HeLa TDP1-KO cells with CPT treatment. Data are represented as mean ± SD. *n* = 6 biologically independent replicates. Two-tailed unpaired *t* test with Welch’s correction was used for statistical analysis. ***p (10 nM CPT) = 0.000121, and ****p (20 nM CPT) = 0.000003. **c** RADAR assay showed increased TOP1cc accumulation in TDP1-KO cells. WT and TDP1-KO cells were either not treated (NT) or treated with 10 μM CPT for 1 h. Double-stranded DNA (dsDNA) is used as a loading control. Data are represented as mean ± SEM. *n* = 8 biologically independent replicates. Two-tailed unpaired *t* test with Welch’s correction was used for statistical analysis. **d** TDP1-KO cells displayed increased DSBs when compared to WT cells after CPT treatment. Representative results of the neutral comet assay were shown (scale bar, 50 µm). Olive tail movement was measured by open comet software and plotted as a box plot. The center line indicates the median, the box bounds indicate the first and third quartiles, and the whiskers indicated the maximum and minimum. Numbers (No.) of cells examined were indicated. Two-tailed unpaired *t* test with Welch’s correction was used for statistical analysis. **e** WT and TDP1-KO cells were treated with 10 µM CPT for the indicated times. Whole-cell extracts were prepared and subjected to Western blotting with the indicated antibodies. Experiments were repeated at least three times, and similar results were obtained. **f** WT, TDP1-KO, and TDP1-KO/TDP1-SFB cells were not treated (NT) or treated with 10 µM CPT for 1 h. Whole-cell extracts were prepared and subjected to Western blotting with the indicated antibodies. Experiments were repeated at least three times, and similar results were obtained.

To investigate the role of TDP1 in the repair of topoisomerase I-mediated DNA damage, we generated TDP1-KO cells using CRISPR-Cas9 genome editing technology in HEK293A and HeLa cells. Consistent with the results of previous studies^16^, both HEK293A and HeLa TDP1-KO cells showed cellular sensitivity to TOP1 poison camptothecin (CPT) (Fig. 1a, b), but not to TOP2 poison etoposide (ETO) (Supplementary Fig. 1a, b), compared to WT cells in the colony formation assay. We repeated the experiments using the Cell-titer Glo assay and obtained similar results (Supplementary Fig. 1c–f). As the activity of TDP1 is specifically involved in the resolution of the covalent tyrosyl-linked TOP1 peptides on DNA, we evaluated this process by RADAR assay using a TOP1 cleavage complex (TOP1cc) antibody^18,19^, which can specifically detect the covalent TOP1-DNA complex in cells. As shown in Fig. 1c, we observed more TOP1cc accumulation in TDP1-KO cells upon CPT treatment compared with that in WT cells.

Besides TDP1, alternative repair factors or pathways have also been suggested to be involved in the repair of TOP1cc^4,16,17^; we evaluated how these factors or pathways are coordinated to process the excess TOP1cc in TDP1-KO cells upon CPT treatment. A neutral comet assay, which detects DSBs at the single cell level, was conducted and revealed that TDP1-KO cells displayed increased DSBs compared to those in WT cells upon CPT treatment (Fig. 1d). Thus, we further examined the DSB-induced DNA damage response (DDR) in WT and TDP1-KO cells upon CPT treatment. We found that two major phosphoinositide 3 kinase–related protein kinases (PI3KKs), ataxia-telangiectasia–mutated (ATM) and DNA-dependent protein kinase catalytic subunit (DNA-PKcs), were hyper-phosphorylated in TDP1-KO cells, either with continuous CPT treatment (Fig. 1e) or with release from CPT treatment (Supplementary Fig. 1g). Similar activation of DDR signaling was observed in HeLa WT and TDP1-KO cells (Supplementary Fig. 2a).

It is known that DNA-PKcs is activated by DSB ends^20^, and ATM is activated by potentially diverse DNA structures, including chromosomal DNA DSBs^20,21^. The observation that these two major DSB-related kinases were hyper-phosphorylated in TDP1-KO cells agrees with the finding of more DSBs in TDP1-KO cells, as detected by a neutral comet assay (Fig. 1d). Moreover, we found that ATM was phosphorylated quickly after CPT treatment in both WT and TDP1-KO cells (Fig. 1e and Supplementary Fig. 2a). On the other hand, the phosphorylation of DNA-PK was delayed and only became obvious 60 min (60’) or more after CPT treatment in both WT and TDP1-KO cells (Fig. 1e and Supplementary Fig. 2a). Moreover, DNA-PK phosphorylation was much more prominent in TDP1-KO cells than ATM phosphorylation, which suggests that the prerequisite for ATM and DNA-PKcs phosphorylation after CPT treatment may be different.

The phosphorylation levels of KAP1 (pKAP1-S824) and H2AX (pH2AX-S139) were also significantly increased in TDP1-KO cells (Fig. 1e and Supplementary Figs. 1g, 2a). The phosphorylation of KAP1 and H2AX occurred rapidly but further increased from 30’ to 60’ after CPT treatment, which coincided with ATM and DNA-PKcs phosphorylation, respectively (Fig. 1e and Supplementary Fig. 2a). On the other hand, an unexpected transient increase and decrease of CHK2 phosphorylation (pCHK2-T68) was observed (Fig. 1e and Supplementary Fig. 2a). CHK2 is a specific substrate of ATM after DNA damage^22^. We were surprised that the phosphorylation level of CHK2-T68 in TDP1-KO cells was not consistent with ATM S1981 phosphorylation (Fig. 1e and Supplementary Figs. 1g, 2a). Time-course experiments showed that while CHK2 was also initially phosphorylated in TDP1-KO cells, its phosphorylation diminished over time (Fig. 1e and Supplementary Fig. 2a). This change was inversely correlated with DNA-PKcs and KAP1 phosphorylation.

We found that when treated with CPT, TDP1-KO cells also displayed increased phosphorylation of CHK1 S345 and RPA32 S4/S8 (Fig. 1g and Supplementary Fig. 2a), which indicate ssDNA accumulation and DNA end resection. When TDP1-KO cells were complemented with ectopically expressed TDP1, nearly all of the observed changes in DDR signaling were restored (Fig. 1f), indicating that hyper-phosphorylation of DNA-PKcs, KAP1, H2AX, and others in TDP1-KO cells after CPT treatment were due to TDP1 loss.

### Proteasome-dependent processing of TOP1cc leads to excess DSB formation in TDP1-KO cells

**Fig. 2:**
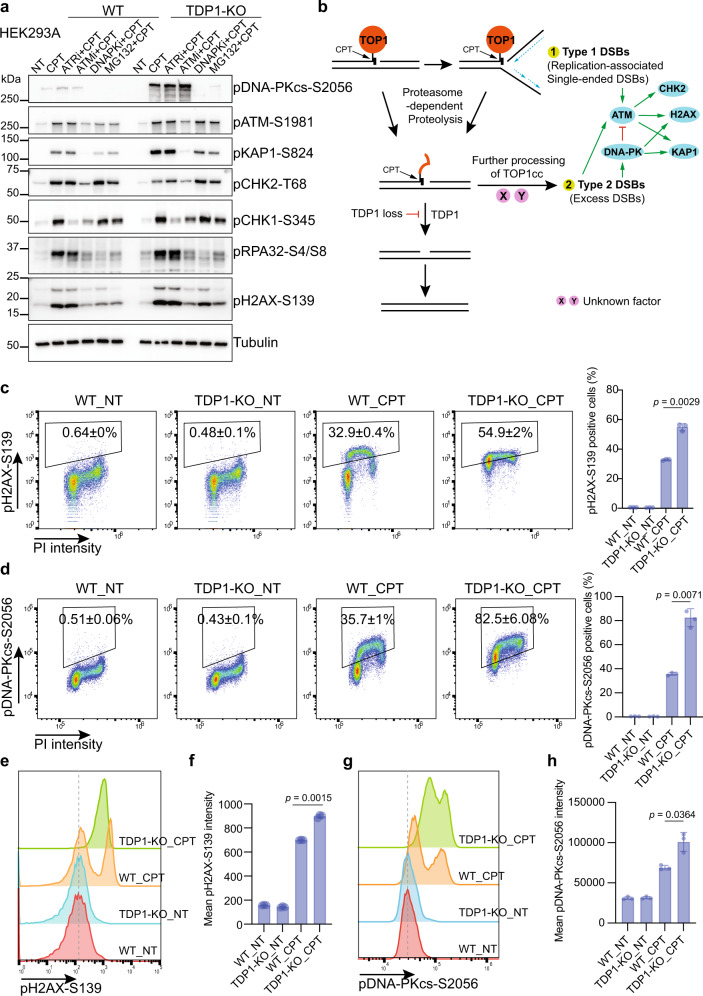
Proteasome-dependent processing of TOP1cc leads to excess DSB formation in TDP1-KO cells. **a** WT and TDP1-KO cells were pre-treated with 10 µM ATRi (AZD6738), 1 µM ATMi (AZD0156), 10 µM DNA-PKi (AZD7648), or 10 µM MG132 for 1 h and then treated with 10 µM CPT for 1 h. Whole-cell extracts were prepared and subjected to Western blotting with the indicated antibodies. Experiments were repeated at least three times, and similar results were obtained. **b** A model of alternative DNA damage signaling and repair in TDP1-KO cells. **c** Flow cytometry analysis of pH2AX-S139 and DNA contents (PI staining) in WT and TDP1-KO cells that were either not treated (NT) or treated with 10 µM CPT for 1 h. Gates for pH2AX-S139-positive cells were shown. Numbers are the percentage of pH2AX-S139-positive cells (mean ± SD). Statistical data were also presented as a bar chart (mean ± SD, *n* = 3 biologically independent experiments). Two-tailed unpaired *t* test with Welch’s correction was used for statistical analysis. **d** as in **c** but pDNA-PKcs-S2056 was analyzed. Statistical data were presented as a bar chart (mean ± SD, *n* = 3 biologically independent experiments). Two-tailed unpaired *t* test with Welch’s correction was used for statistical analysis. **e** A flow cytometry analysis of pH2AX-S139 intensity in WT and TDP1-KO cells that were either not treated (NT) or treated with 10 µM CPT for 1 h. **f** Quantification of **e**. Mean pH2AX-S139 intensity were shown in a bar chart (mean ± SD, *n* = 3 biologically independent experiments). Two-tailed unpaired *t* test with Welch’s correction was used for statistical analysis. **g** as in **e** but pDNA-PKcs-S2056 intensity was analyzed. **h** Quantification of **g**. Mean pDNA-PKcs-S2056 intensity was shown in a bar chart (mean ± SD, *n* = 3 biologically independent experiments). Two-tailed unpaired *t* test with Welch’s correction was used for statistical analysis.

To further characterize the DSB-induced DDR signaling in TDP1-KO cells, we pretreated cells with inhibitors of ATR (AZD6738), ATM (AZD0156), DNA-PK (AZD7648), or proteasome (MG132) before CPT treatment. The phosphorylation of KAP1-S824 and H2AX-S139 decreased when cells were treated with either DNA-PK inhibitor (DNAPKi) or ATM inhibitor (ATMi), but not with ATR inhibitor (ATRi) (Fig. 2a and Supplementary Fig. 2b). On the other hand, ATRi treatment reduced CHK1-S345 phosphorylation (Fig. 2a and Supplementary Fig. 2b). These data indicate that multiple DDR signaling pathways are activated after CPT treatment. However, the most notable differences between WT and TDP1-KO are DNA-PKcs-S2056, KAP1-S824, and H2AX-S139 phosphorylation, which depend largely on DNA-PK and to a lesser degree on ATM.

Interestingly, we showed that DNA-PK hyperphosphorylation is inversely corelated with CHK2-T68 phosphorylation (Fig. 1e), indicating that DNA-PK activation somehow inhibits ATM-dependent CHK2 phosphorylation. Indeed, an early study reported that DNA-Pkcs can directly phosphorylate ATM at multiple sites and hence inhibit ATM kinase activity^23^. In agreement with this hypothesis, we noted that the phosphorylation of CHK2-T68 increased when cells were treated with DNA-PK inhibitor (Fig. 2a and Supplementary Fig. 2b). This inhibitory effect of DNA-PKcs on the phosphorylation of CHK2 was also observed after IR treatment (Supplementary Fig. 2c). However, we did not observe any change in ATM-S1981 phosphorylation, which is an autophosphorylation site of ATM^24,25^. Moreover, as pCHK2-T68 induced by DNA damage is found in soluble fractions, but not in the chromatin-enriched fraction^26^, we separated the soluble and chromatin-bound fractions. We found that ATM-S1981 phosphorylation in TDP1-KO cells was higher than that in WT cells in both fractions (Supplementary Fig. 2d), which excludes the possibility that a change in pATM localization causes reduced CHK2 phosphorylation.

When cells were treated with proteasome inhibitor MG132, we noticed a slight reduction in ATM-S1981 phosphorylation but a nearly complete inhibition of DNA-PKcs S2056 phosphorylation (Fig. 2a and Supplementary Fig. 2b). As mentioned above, we speculate that the prerequisite for ATM and DNA-PKcs phosphorylation after CPT treatment may be different. So, it is highly possible that there are two types of DSBs that are formed upon treatment with TOP1 poison CPT (Fig. 2b). TOP1cc may encounter replication forks and be converted into single-ended DSBs, which activates ATM. The activation of ATM occurs immediately following CPT treatment and does not depend on the proteolysis of covalently bound TOP1. The proteasome-dependent degradation of the covalently bound TOP1 makes it accessible to TDP1, which directly removes the proteolyzed TOP1cc. However, when TDP1 is lost, the proteolyzed TOP1cc cannot be efficiently repaired; this promotes further processing of these lesions, which produces a different type of DSBs, named type 2 DSBs or excess DSBs, that can lead to the activation of DNA-PK and its downstream phosphorylation events. As mentioned above, H2AX and KAP1 are substrates of ATM and DNA-PKcs after TOP1-induced DNA damage, which can serve as markers for total DSB signaling after CPT treatment. DNA-PKcs can only be activated by DSBs generated after TOP1 degradation and can be used as a marker to illustrate the formation of excess DSBs at the later step.

**Fig. 3:**
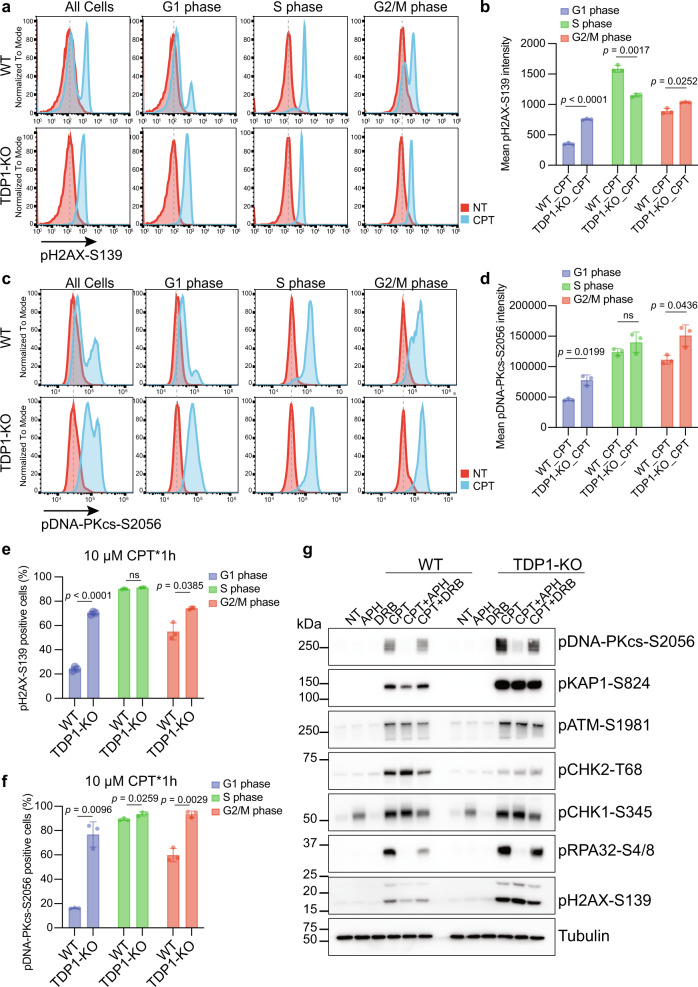
Excess DSBs formed in TDP1-KO cells after CPT treatment were not cell cycle coordinated. **a** Flow cytometry analysis of pH2AX-S139 intensity in WT and TDP1-KO cells either not treated (NT) or treated with 10 µM CPT for 1 h. Cells from G1, S, or G2/M phase were independently presented. **b** Quantification of **a**. Mean pH2AX-S139 intensity from three independent experiments are shown in a bar chart (mean ± SD). Two-tailed unpaired *t* test with Welch’s correction was used for statistical analysis. **c** Flow cytometry analysis of pDNA-PKcs-S2056 intensity in WT and TDP1-KO cells either NT or treated with 10 µM CPT for 1 h. Cells from G1, S, or G2/M phase were independently presented. **d** Quantification of **c**. Mean pDNA-PKcs-S2056 intensity from three independent experiments are shown in a bar chart (mean ± SD). Two-tailed unpaired *t* test with Welch’s correction was used for statistical analysis. **e** Percentage of pH2AX-S139-positive cells from three independent experiments are shown in a bar chart (mean ± SD). Two-tailed unpaired *t* test with Welch’s correction was used for statistical analysis. **f** Percentage of pDNA-PKcs-S2056-positive cells from three independent experiments are shown in a bar chart (mean ± SD). Two-tailed unpaired *t* test with Welch’s correction was used for statistical analysis. **g** WT and TDP1-KO cells were pre-treated with 1 µM APH, or 200 µM DRB for 1 h and then treated with 10 µM CPT for 1 h. Whole-cell extracts were prepared and subjected to Western blotting with the indicated antibodies. Experiments were repeated at least three times, and similar results were obtained.

CPT-induced and replication-associated DSBs are likely single-ended DSBs, which should be generated mainly in S phase. However, we do not yet know exactly how the processed DSBs are generated following proteasome-dependent degradation of TOP1cc and whether these DSBs are created exclusively in S phase cells. To better characterize the formation of these two types of TOP1-induced DSBs, we used fluorescence-activated cell sorting analyses to detect total DSB signaling with pH2AX-S139 antibody and the signaling from the processed DSBs with pDNA-PKcs-S2056 antibody. After CPT treatment, the percentage of pH2AX-S139- and pDNA-PKcs-S2056-positive cells (Fig. 2c, d) and the mean pH2AX-S139 and pDNA-PKcs-S2056 intensity (Fig. 2e–h) increased in both WT and TDP1-KO cells, with a significantly higher increase in TDP1-KO cells than that in WT cells, which agree with our Western blot results (Fig. 1e). The cell cycle distribution before and after CPT treatment in WT and TDP1-KO cells did not change much (Supplementary Fig. 3c). Increased pH2AX-S139 and pDNA-PKcs-S2056 signals were mostly noticeable in S phase WT cells after CPT treatment; however, these signals appeared in all cell cycle phases in TDP1-KO cells (Fig. 2c, d). When independently comparing the pH2AX-S139 and pDNA-PKcs-S2056 signals from different cell phases in WT and TDP1-KO cells after CPT treatment, we found that in G1 and G2/M phase cells, the mean pH2AX-S139 and pDNA-PKcs-S2056 intensity (Fig. 3a–d) and the percentage of pH2AX-S139- and pDNA-PKcs-S2056-positive cells (Fig. 3e, f) were higher in TDP1-KO cells than in WT cells. As nearly all of the S phase cells in WT cells have been pH2AX-S139- and pDNA-PKcs-S2056-positive (Fig. 3e, f), which suggests that the CPT concentration we used saturated the DDR response in S phase. This may be the reason that we did not see much difference in the DDR response signal between the S phase TDP1-KO cells and WT cells after CPT treatment (Fig. 3a–f). Therefore, the TOP1cc-converted DSBs were generated from G1, G2/M, and perhaps S phase cells.

Processing of CPT poisoned TOP1 into DNA damage by replication and transcription has been suggested^4,27–30^. We thus examined how inhibition of DNA replication or transcription would affect DSB generation after CPT treatment, especially for the excess DSBs generated after the proteasome-mediated degradation of TOP1cc in TDP1-KO cells. We pre-treated cells with APH, an inhibitor of DNA polymerase alpha, or 5, 6- DRB, a transcription inhibitor, before treating cells with CPT. We found that inhibition of replication by APH significantly decreased the phosphorylation of DNA-PKcs in both WT and TDP1-KO cells (Fig. 3g). Inhibition of transcription by pre-treatment with DRB also modestly decreased the phosphorylation of DNA-PKcs in WT and TDP1-KO cells (Fig. 3g). These results suggest that the formation of excess DSBs depends on DNA replication and to a lesser degree on transcription. However, since inhibition of DNA replication or transcription also has other effects that may influence the proteolysis of TOP1cc, we cannot conclude a direct role of DNA replication and transcription in converting TOP1cc into excess DSBs.

### Genome-wide CRISPR/Cas9 screens in wild-type and TDP1-KO cells with TOP1 poison

As discussed above (Fig. 2b), we speculate that the accumulated TOP1cc in TDP1-KO cells is processed and converted into DSBs for further repair, which may compensate for the loss of TDP1. Thus, inhibition of this process or its downstream repair pathways may exacerbate the response of TDP1-KO cells to CPT treatment. To uncover potential repair factors that participate in this process, we launched unbiased whole genomic screening in both WT and TDP1-KO cells, with or without TOP1 poison CPT. Screenings were performed as previously described^31^. After deep sequencing and obtaining sgRNA counts in each sample (Supplementary Data 1 and 2), we used a drug Z analysis to compare the CPT-treated group with the NT group in WT or TDP1-KO cells to determine CPT sensitivity profiling in each cell line (Supplementary Data 3 and 4).

**Fig. 4:**
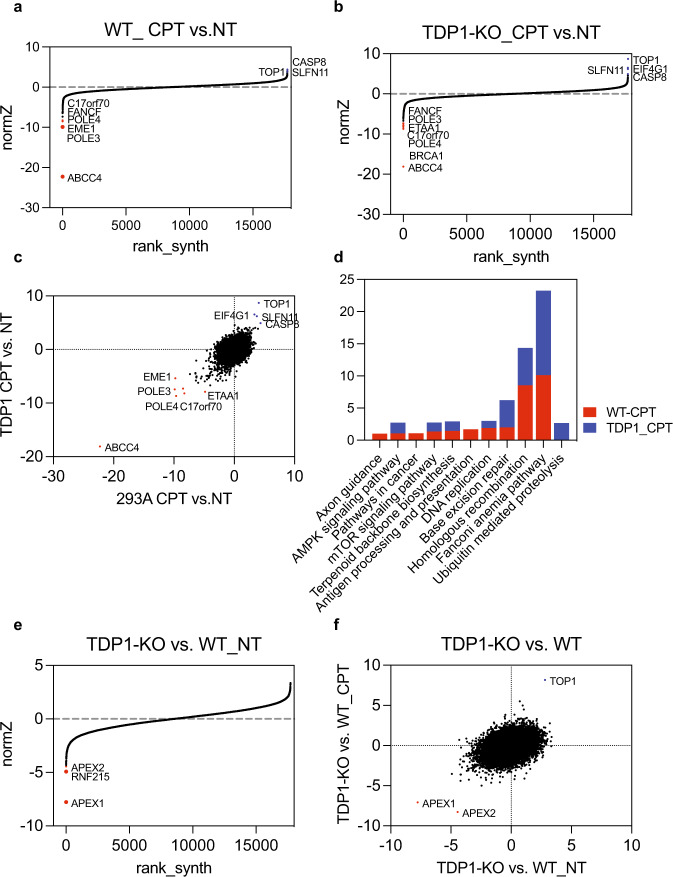
Genome-wide CRISPR/Cas9 screens in WT and TDP1-KO cells with TOP1 poison. **a** Ranking of CPT co-essential genes on the basis of drug Z analysis of the results of CRISPR/Cas9-based screening in HEK293A-WT cells. The z-score was used to define a possible synthetic lethal interaction with CPT. All genes targeted by the Toronto Knock Out Library v3 were scored according to the fold change of levels of their sgRNAs. The CPT-treated group and not treated (NT) group in WT cells were compared. Genes whose loss of function led to CPT sensitivity appear on the left side, with a minus Z score, and genes whose loss of function led to CPT resistance appear on the right side, with a positive Z score. Top-ranked genes on either side are marked. **b** Ranking of CPT co-essential genes on the basis of a drug Z analysis of the results of CRISPR/Cas9-based screening in TDP1-KO cells. Data were analyzed and presented as in **a**. The CPT-treated group and NT group in TDP1-KO cells were compared. **c** Combinational comparison of CPT co-essential genes between HEK293A-WT and TDP1-KO cells. The z-scores from **a**, **b** were used. **d** A *p* value is calculated from the normZ and corrected for multiple hypothesis testing using the method of Benjamini and Hochberg. KEGG pathway analysis of genes whose loss of function led to CPT sensitivity (*p* < 0.01) in WT and TDP1-KO cells was performed with DAVID Bioinformatics Resources 6.8, NIAID/NIH (https://david.ncifcrf.gov/summary.jsp). **e** Ranking of TDP1 co-essential genes on the basis of a drug Z analysis of the results of CRISPR-/Cas9-based screening. The z-score was used to define a possible synthetic lethal interaction with TDP1. All genes targeted by the Toronto Knock Out Library v3 were scored according to the fold change of their sgRNA levels. The NT TDP1-KO cell group and NT WT cell group were compared. **f** A combinational comparison was performed of TDP1 co-essential genes between HEK293A-WT and TDP1-KO cells using the z-score of the NT TDP1-KO cell group compared to the NT WT group and the CPT-treated TDP1-KO group compared to the CPT-treated WT group.

As presented in Fig. 4a, b, the CPT sensitivity profiles of WT and TDP1-KO cells were generally very similar, with ABCC4 as the top-ranked gene that sensitizes both WT and TDP1-KO cells to CPT treatment and TOP1 and SLNF11 as the top genes that confer CPT resistance. ABCC4 is an ATP-binding cassette (ABC) transporter that is capable of pumping a wide variety of molecules across the membrane^32^; it is also called MRP4 and belongs to the multi-drug resistance subfamily^32^. Frequent overexpression of ABCC4 has been observed in primary neuroblastoma and ovarian cancer and is significantly associated with a poor clinical prognosis^33,34^. Overexpression of ABCC4 in vitro has been shown to confer resistance to CPT and its orthologues^33,35^. ABCC4 is expressed in multi-tissues (Supplementary Fig. 4a). HEK293A is a transformed cell line that was established from primary embryonal human kidneys with relatively high ABCC4 expression (Supplementary Fig. 4a), which may be the reason that it ranks as the top gene whose loss sensitizes these cells to CPT treatment. Our screening results provide strong genetic evidence that the ABCC4 expression level can be used as an important marker for evaluating cancer cells’ response to CPT treatment.

Besides ABCC4, POLE3, POLE4, and the Fanconi anemia pathway genes FANCF and C17orf70 are top candidates that sensitize both wild-type and TDP1-KO cells to CPT treatment (Fig. 4a–c). A KEGG functional pathway analysis of CPT-sensitive genes with *p* value < 0.01 showed that the Fanconi anemia pathway, homologous recombination, and base excision repair are the top three pathways that determine cellular sensitivity to CPT treatment (Fig. 4d and Supplementary Data 5). In TDP1-KO cells, the depletion of genes that are involved in the ubiquitin-mediated proteolysis pathway made cells more sensitive to CPT (Fig. 4d and Supplementary Data 5), which suggests that repair pathways that function in the absence of TDP1 depend on the ubiquitin-mediated proteolysis pathway.

Although CPT has been widely used as an effective anticancer drug, resistance is still a critical clinical problem. Our screening identified several potential gene candidates whose loss confers cellular CPT resistance (Fig. 4a–c). Two known factors are TOP1 and SLNF11. CPT is known to target TOP1 as its mechanism of action. Previous studies in mammalian cells and yeast have already suggested that mutations of TOP1 confers resistance to CPT and that overexpression of TOP1 could result in increased CPT sensitivity^36^. SLFN11 was previously discovered in a genome-wide analysis that showed a positive correlation with the response to DNA-damage agents, including CPT^37,38^. Besides these two genes, CASP8 and EIF4G1 also ranked as top candidates that confer resistance to CPT. CASP8 is a member of the caspase family that is involved in apoptosis and is a favorable prognosis marker for ovarian cancer^39^. The survival duration of patients with tumors showing low CASP8 expression was shorter than that of higher CASP8 expression (Supplementary Fig. 4b). We speculate that its loss may prevent apoptosis caused by TOP1-induced damage and therefore show resistance to CPT treatment. EIF4G1 is a member of the eIF4F complex. Increased expression of EIF4G1 has been found to selectively increase the translation of mRNAs that are involved in cell survival and DDR^40^. However, it remains unclear how EIF4G1 loss promotes CPT resistance, which needs further investigation.

We compared the screening results of WT and TDP1-KO cells, with or without CPT treatment, and found that the depletion of most genes caused a similar effect in both wild-type and TDP1-KO cells (Fig. 4c), suggesting that the effect caused by the drug is dominant over TDP1 depletion.

### APEX1 and APEX2 are synthetic lethal with TDP1

To identify factors that accommodate TDP1 deficiency, with or without TOP1-induced damage, we performed drug Z analyses by comparing the TDP1-KO NT group with the WT NT group to obtain synthetic lethality profiling with loss of TDP1 (Supplementary Data 6). Similarly, we also compared the TDP1-KO CPT-treated group with the WT CPT-treated group to obtain CPT-dependent synthetic lethality profiling with loss of TDP1 (Supplementary Data 7). Interestingly, we found that loss of APEX1 and APEX2 showed synthetic lethality with TDP1 deficiency, with or without CPT treatment (Fig. 4e, f). These results agree with the data in RPE1 cells reported by Dr. Daniel Durocher and colleagues^41^ and indicate that the synthetic lethality between TDP1 and APEX1/2 is a common mechanism in different cell lines. By analyzing the normalized fold change of the four sgRNAs that target APEX1 or APEX2, we found that the co-lethality between TDP1 and APEX1 was comparable in the CPT-treated and NT groups (Supplementary Fig. 4c). However, APEX2/TDP1 double-deficient cells showed mild synthetic lethality without any treatment, and the synthetic lethality is magnified with CPT treatment (Supplementary Fig. 4d), which agrees with the finding that APEX2 can process 3’ blocked TOP1cc in the absence of TDP1^41^.

**Fig. 5:**
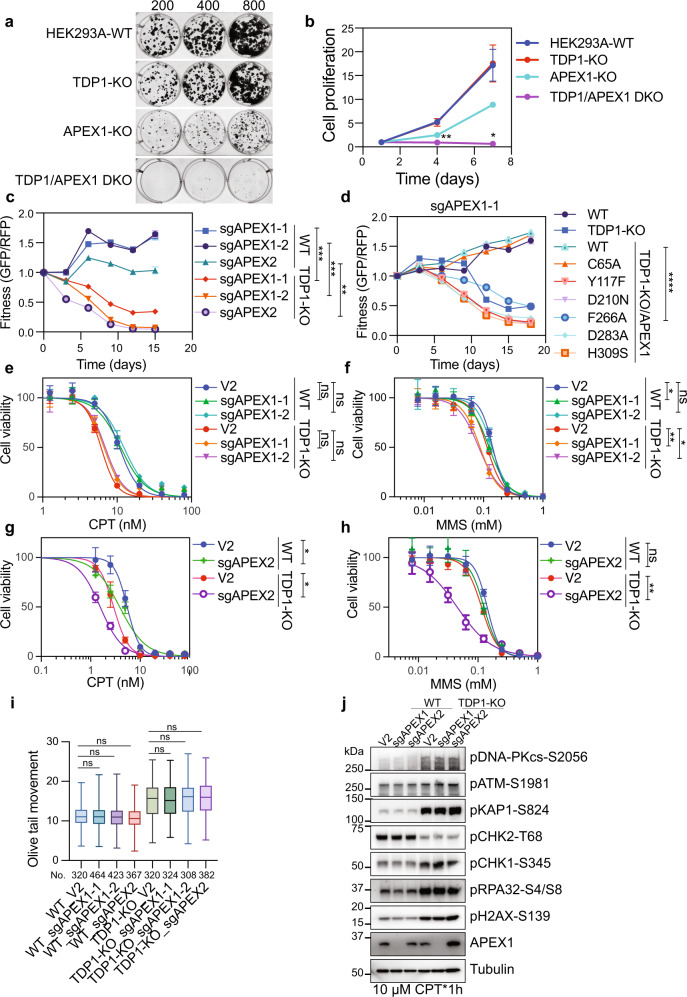
APEX1 and APEX2 are synthetic lethal with TDP1. **a** The relative cell growth rates of indicated cell lines were detected by colony formation assay. **b** The relative cell growth rates of indicated cell lines were detected by a Cell-Titer Glo assay. Data are represented as mean ± SD (*n* = 3 biologically independent experiments). Two-tailed unpaired *t* test with Welch’s correction was used for statistical analysis between TDP1-KO and TDP1/APEX1-DKO. **p (day 4) = 0.0056 and *p (day 7) = 0.0171. **c** Competitive growth assays in WT and TDP1-KO cells infected with a virus expressing the indicated sgRNAs. Data are presented as mean ± SD (*n* = 3 biologically independent experiments). Two-tailed unpaired *t* test with Welch’s correction was used for statistical analysis. ***p (WT_sgAPEX1-1 vs. TDP1-KO_sgAPEX1-1) = 0.0008, ***p (WT_sgAPEX1-2 vs. TDP1-KO_sgAPEX1-2) = 0.0005, and **p (WT_sgAPEX2 vs. TDP1-KO_sgAPEX2) = 0.0012. **d** Competitive growth assays in cells infected with a virus expressing sgAPEX1-1 in WT, TDP1-KO cells, or TDP1-KO cells overexpressing an sgRNA-resistant wild-type (WT) or mutation APEX1. Data are presented as mean ± SD (*n* = 3 biologically independent experiments). Two-tailed unpaired *t* test with Welch’s correction was used for statistical analysis. ****p (TDP1-KO/APEX1-WT vs. TDP1-KO/APEX1-F266) = 0.000058. **e**, **f** WT and TDP1-KO cells were infected with pLenti-V2 empty vector or pLenti-V2-sgRNAs targeting APEX1. Cell proliferation was measured using a CellTiter-Glo assay after 3 days in the presence of the indicated concentrations of CPT (**e**) or MMS (**f**). Data are presented as the mean ± SD (*n* = 3 biologically independent experiments). Two-tailed unpaired *t*-test was used for statistical analysis of the IC50 of each cell line. *p (MMS: WT_V2 vs. WT_sgAPEX1-1) = 0.017755, **p (MMS: TDP1-KO_V2 vs. TDP1-KO_sgAPEX1-1) = 0.004144, *p (MMS: TDP1-KO_V2 vs. TDP1-KO_sgAPEX1-2) = 0.011301, and ns not significant. **g**, **h** as in **e**, **f** but using an sgRNA targeting APEX2. Two-tailed unpaired *t*-test was used for statistical analysis of the IC50 of each cell line. *p (CPT: WT_V2 vs. WT_sgAPEX2) = 0.013051, *p (CPT: TDP1-KO_V2 vs. TDP1-KO_sgAPEX2) = 0.021743, **p (MMS: TDP1-KO_V2 vs. TDP1-KO_sgAPEX2) = 0.001097, and ns not significant. **i** WT and TDP1-KO cells were infected with pLenti-V2 empty vector or pLenti-V2-sgRNAs targeting APEX1 or APEX2. A neutral comet assay was performed after treating cells with 1 µM CPT for 1 h. Olive tail movement was measured by open comet software and plotted as a box plot. The center line indicates the median, the box bounds indicate the first and third quartiles, and the whiskers indicate maximum and minimum. Numbers (No.) of cells examined were indicated. A one-way ANOVA Kruskal–Wallis test was used for statistical analysis. ns, not significant. **j** WT and TDP1-KO cells were infected with pLenti-V2 empty vector or pLenti-V2-sgRNAs targeting APEX1 or APEX2. Cells were then treated with 10 µM CPT for 1 h. Whole-cell extracts were prepared and subjected to Western blotting with the indicated antibodies. Experiments were repeated at least three times, and similar results were obtained.

We further validated these findings by transfecting TDP1-KO cells with sgRNAs targeting APEX1. Single clones were then separated and assessed by Western blotting to determine APEX1 protein expression. Initially, we were able to obtain several clones with co-depletion of TDP1 and APEX1 expression; however, these clones grew very slowly, and they kept dying, and hardly formed colonies (Fig. 5a, b), which confirmed the co-lethality between TDP1 and APEX1. We also examined DNA damage checkpoint signaling in these cells and found that CHK2 phosphorylation was specifically increased in DKO cells (Supplementary Fig. 5a), indicating that more DSB formation is the cause of the extremely slow proliferation observed in these DKO cells. Although we were able to isolate these DKO clones of TDP1 and APEX1, the cells died after several weeks. We also confirmed the synthetic lethality between APEX1/APEX2 and TDP1 with a competitive growth assay and observed that sgRNAs targeting APEX1 resulted in loss of fitness in TDP1-KO cells, but not in WT cells (Fig. 5c). sgRNA targeting APEX2 resulted in some loss of fitness in WT cells but lost more viability in TDP1-KO cells (Fig. 5c). We obtained similar results from a competitive growth assay in HeLa cells (Supplementary Fig. 5b).

As APEX1 has both redox and DNA repair enzymatic activities, we treated TDP1-KO cells with either APEX1 redox inhibitor or APEX1 enzymatic inhibitor. TDP1-KO cells were not sensitive to APEX1 redox inhibitor but did show slight sensitivity to APEX1 enzymatic inhibitor (Supplementary Fig. 5c, d). Furthermore, prior introduction of an sgRNA-resistant wild type or redox activity mutation (C65A)^42^ APEX1 transgene, but not a transgene expressing enzymatic inactive APEX1 (Y117F, D210N, F266A, D283A, or H309S)^43^, rescued APEX1-TDP1 synthetic lethality (Fig. 5d), indicating that the enzymatic activity of APEX1 is essential in the TDP1-KO background. APEX1 has both AP-endonuclease and 3’ to 5’ exonuclease activity. The AP-endonuclease activity cleaves at AP sites, while the exonuclease activity excises bulkier 3’ blocks. As the two activities used a single active site, we could not separate them by mutations. We then transfected WT and TDP1-KO cells with sgRNAs targeting APEX1 and monitored the cellular sensitivity to CPT or alkylating agent MMS, which causes base damage. Loss of APEX1 did not show any sensitivity to CPT treatment in both WT and TDP1-KO cells (Fig. 5e) but decreased cell viability following exposure to MMS, and this sensitivity was enhanced upon TDP1 deficiency (Fig. 5f). Together, these results suggest that APEX1 and TDP1 carry out partly redundant activities in repairing base damage, which has been inferred previously^44^.

The co-deficiency of TDP1 and APEX2 are synthetic lethal in RPE1 cells: TDP1 and APEX2 remove TOP1cc in distinct cellular contexts^41^. We confirmed the synthetic lethality between APEX2 and TDP1 with a competitive assay in both HEK293A and HeLa cells (Fig. 5c and Supplementary Fig. 5b). We then transfected WT and TDP1-KO cells with sgRNAs targeting APEX2 and monitored the cellular sensitivity to CPT or MMS. We found that deficiency of APEX2 has an additive effect to CPT and MMS treatment in TDP1-KO cells (Fig. 5g, h), which suggested that APEX2 and TDP1 have redundant roles in both base damage repair and TOP1-induced DNA damage repair.

We determined whether APEX2 contributes to the conversion of accumulated TOP1cc into excess DSBs in TDP1-KO cells. As APEX2 showed the ability to process the TOP1cc mimicking structure in vitro^41^, we first monitored the accumulation of TOP1cc by RADAR assay in cells transfected with sgRNA targeting APEX2 after CPT treatment. Transfecting of cells with sgRNA targeting APEX2 slightly increased the TOP1cc accumulation in WT cells (Supplementary Fig. 5e). However, we did not observe any additive effect on the TOP1cc accumulation with TDP1 and APEX2 co-deficiency. As our RADAR assay showed variations (Fig. 1c), we cannot exclude the possibility that APEX2 can promote TOP1cc removal with TDP1 loss. We next checked the DSB formation by neutral comet assay. As shown in Fig. 5i, transfecting cells with sgRNA targeting APEX1 or APEX2 did not reduce the formation of DSBs in both WT and TDP1-KO cells after CPT treatment. Correspondingly, there is no change in DSB-induced DDR signaling with APEX1 or APEX2 deficiency in WT and TDP1-KO cells after CPT treatment (Fig. 5j). Thus, APEX1 and APEX2 are not involved in the conversion of TOP1cc into excess DSBs with TDP1 deficiency.

### Excision pathways that work in parallel with TDP1 in removing TOP1cc are not required for the excess DSB generation with TDP1 loss

TOP1cc can also be resolved by nucleases that cleave on the DNA strand to which TOP1 was covalently bound^17,45^. Multiple excision pathways have been reported to work in parallel with TDP1 in removing TOP1cc after CPT treatment^4,16,17^. We then determined whether the excess DSBs generated in TDP1-KO cells are by-products of the function of these excision pathways.

**Fig. 6:**
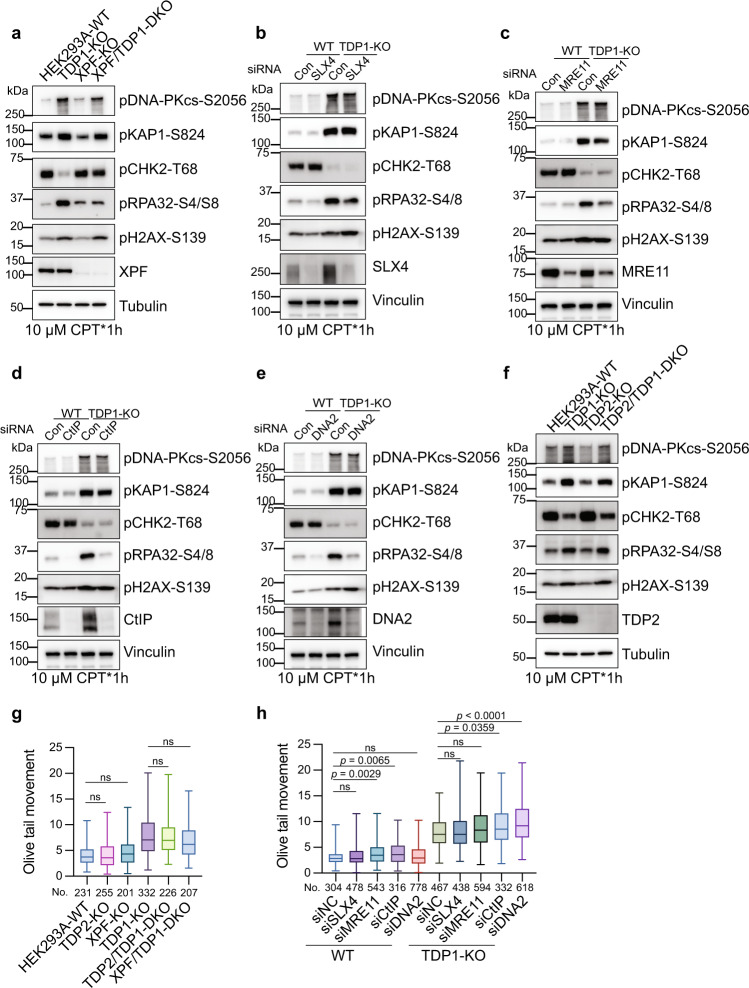
Excision pathways that work in parallel with TDP1 in removing TOP1cc are not required for excess DSB generation with TDP1 loss. **a** HEK293A-WT, TDP1-KO, XPF-KO, and XPF/TDP1-DKO cells were treated with 10 µM CPT for 1 h. Whole-cell extracts were prepared and subjected to Western blotting with the indicated antibodies. Experiments were repeated at least three times, and similar results were obtained. **b**–**e** Control siRNA or siRNA against SLX4 (**b**), MRE11 (**c**), CtIP (**d**), or DNA2 (**e**) were transfected into WT or TDP1-KO cells. 72 h after siRNA transfection, cells were treated with 10 µM CPT for 1 h. Whole-cell extracts were prepared and subjected to Western blotting with the indicated antibodies. Experiments were repeated at least three times, and similar results were obtained. **f** HEK293A-WT, TDP1-KO, TDP2-KO, and TDP2/TDP1-DKO cells were treated with 10 µM CPT for 1 h. Whole-cell extracts were prepared and subjected to Western blotting with the indicated antibodies. Experiments were repeated at least three times, and similar results were obtained. **g** A neutral comet assay was performed after treating the indicated cells with 1 µM CPT for 1 h. Olive tail movement was measured by open comet software and plotted as a box plot. The center line indicates the median, the box bounds indicate the first and third quartiles, and the whiskers indicate maximum and minimum. Numbers (No.) of cells examined were indicated. A one-way ANOVA Kruskal–Wallis test was used for statistical analysis. ns, not significant. **h** Control siRNA or siRNA against SLX4, MRE11, CtIP, or DNA2 were transfected into WT or TDP1-KO cells. 72 h after siRNA transfection, a neutral comet assay was performed after treating cells with 1 µM CPT for 1 h. Olive tail movement was measured by open comet software and plotted as a box plot. The center line indicates the median, the box bounds indicate the first and third quartiles, and the whiskers indicate the maximum and minimum. Numbers (No.) of cells examined were indicated. A one-way ANOVA Kruskal–Wallis test was used for statistical analysis. ns not significant.

Structure-specific nuclease ERCC1-XPF and its yeast ortholog Rad10-Rad1 are a 3’ flap endonuclease complex that is involved in the nucleotide excision repair pathway; they have been shown to participate in the repair of TOP1-induced DNA damage, potentially by removing the oligonucleotides that contain a tyrosyl-phosphodiester bond on their 3’ or that directly cut the 3’-phosphotyrosyl bonds^46,47^. We generated an XPF-deficient cell line, in which only an extremely low level of XPF was expressed, and cells showed strong DNA-crosslink repair defects^48^. XPF-deficient cells showed mild cellular sensitivity to CPT treatment (Supplementary Fig. 6a). Co-depletion of TDP1 and XPF resulted in slightly greater cellular sensitivity than did depletion of TDP1 alone (Supplementary Fig. 6a). We observed a mild reduction in the pDNA-PKcs-S2056 signal in XPF and TDP1 co-deficiency cells (Fig. 6a). However, we previously showed that XPF deficiency caused a slower cell growth rate^48^. We cannot exclude the possibility that the mild effect of XPF on DSB-induced DDR signaling may not be due to a direct role in DSB generation but to the slower DNA replication rate in these XPF-KO cells. In contrast, a significant reduction of pRPA32-S4/S8 signal was observed in XPF and TDP1 co-deficiency cells after CPT treatment (Fig. 6a). Another endonuclease, SLX4-SLX1, has also been shown to work in a parallel pathway with TDP1 in removing TOP1cc^45^. When transfecting cells with siRNA targeting SLX4, we observed no DSB-induced pDNA-PKcs-S2056, pKAP1-S824, or pH2AX-S139 signal change (Fig. 6b). Similar to XPF, knocking down SLX4 also decreased the pRPA32-S4/S8 level after CPT treatment, which suggested that SLX4 contributes to the ssDNA generation during the repair process (Fig. 6b). Thus, XPF and SLX4 may be involved in the repair of TOP1-induced DNA damage in TDP1-deficient cells. However, these repair factors do not contribute significantly to the excess DSB generation in TDP1-KO cells.

We then investigated the nucleases that are involved in DSB end resection. MRE11, together with CtIP, initiate DSB end resection and generate a short-ranged end resection of the DSB ends^49^. Opposite functions of MRE11 and CtIP in the removal of TOP1cc have been reported, with MRE11 promoting the removal of TOP1cc and CtIP inhibits it^45,50–52^. We observed no obvious pDNA-PKcs-S2056, pKAP1-S824, and pH2AX-S139 signal change when knocking down either MRE11 or CtIP (Fig. 6c, d). On the other hand, a significant reduction in the pRPA32-S4/S8 signal was observed with either MRE11 or CtIP deficiency (Fig. 6c, d). When knocking down DNA2, another nuclease responsible for long-ranged DSB end resection, similar phenotypes were observed (Fig. 6e). These results suggest that the major contribution of MRE11 and CtIP to the repair process of TOP1-induced damage is to coordinate DNA end resection.

Tyrosyl DNA phosphodiesterase-2 (TDP2) is not a nuclease, but it has both strong 5’-tyrosyl DNA phosphodiesterase (5’-TDP) activity and weak 3’-tyrosyl DNA phosphodiesterase (3’-TDP) activity in vitro^53,54^, which has also been shown to promote repair of TOP1-mediated DNA damage in the absence of TDP1 in Avian DT40 and murine cells^53^. We generated TDP1/TDP2–double knockout (DKO) cells by CRISPR-Cas9. As expected, TDP2-KO cells were hyper-sensitive to TOP2 poison (ETO) (Supplementary Fig. 6b–e, right panel). On the other hand, inconsistent with findings reported in the literature, we observed mild cellular sensitivity of TDP2-KO cells to CPT and no additive effect with TDP1 deficiency (Supplementary Fig. 6b–e, left panel). We carefully repeated the results in both HEK293A and HeLa cells using a colony formation assay and Cell-titer Glo assay and obtained very similar results (Supplementary Fig. 6b–e). One possible explanation for the observed inconsistence is that the expression and activities of TDP1 and TDP2 in removing 3’-phosphotyrosyl and 5’-phosphotyrosyl bonds may be different in different cell lines. Nevertheless, depletion of TDP2 did not have much effect on DSB-induced DDR signaling activation in TDP1-KO cells after CPT treatment (Fig. 6f), indicating that TDP2 is not required for the excess DSB generation in TDP1-KO cells.

We directly checked the amount of CPT-induced DSBs by neutral comet assay. As shown in Fig. 6g, h, the deficiency of XPF, SLX4, MRE11, CtIP, and DNA2 did not reduce the amount of CPT-induced DSB formation in TDP1-KO cells. Depletion of TDP2 and XPF did not change CPT-induced DSB formation in both WT and TDP1-KO cells (Fig. 6g). When knocking down MRE11, CtIP, and DNA2, CPT-induced DSBs in WT and TDP1-KO cells somehow increased (Fig. 6h), which indicates an important role for MRE11, CtIP, and DNA2 in repairing the DSBs formed after TOP1 poison by CPT.

As XPF, SLX4, MRE11, CtIP, and TDP2 have all been suggested to function in parallel with TDP1 in resolving TOP1cc, we also checked the TOP1cc accumulation in cells with deficiency of these genes. Unfortunately, we failed to detect any significant accumulation of TOP1cc with deficiency of any of these genes (Supplementary Fig. 7a, b). Even though a trend in accumulation of TOP1cc was observed in XPF-, MRE11-, and CtIP-deficient cells, no further additive effect was observed with co-depletion of TDP1 (Supplementary Fig. 7a, b). As mentioned above, this technical issue may prevent us from detecting their contributions in removing TOP1cc.

### MUS81 is required for conversion of accumulated TOP1cc into excess DSBs after CPT treatment

**Fig. 7:**
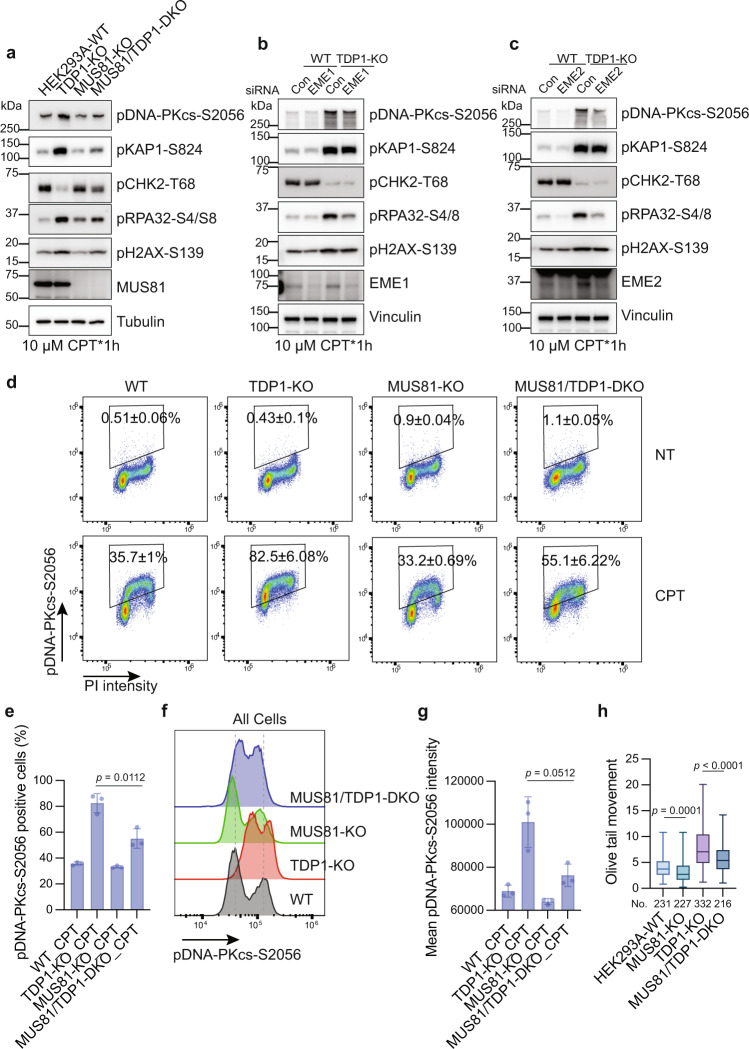
MUS81 is required for conversion of accumulated TOP1cc into excess DSBs after CPT treatment. **a** HEK293A-WT, TDP1-KO, MUS81-KO, and MUS81/TDP1-DKO cells were treated with 10 µM CPT for 1 h. Whole-cell extracts were prepared and subjected to Western blotting with the indicated antibodies. Experiments were repeated at least three times, and similar results were obtained. **b**, **c** Control siRNA or siRNA against EME1 (**b**) or EME2 (**c**) were transfected into WT or TDP1-KO cells. 72 h after siRNA transfection, cells were treated with 10 µM CPT for 1 h. Whole-cell extracts were prepared and subjected to Western blotting with the indicated antibodies. Experiments were repeated at least three times, and similar results were obtained. **d** Flow cytometry analysis of pDNA-PKcs-S2056 and DNA contents (PI staining) in WT, TDP1-KO, MUS81-KO, and MUS81/TDP1-DKO cells either not treated (NT) or treated with 10 µM CPT for 1 h. Gates for pDNA-PKcs-S2056-positive cells are shown. The presented numbers are the percentage of pDNA-PKcs-S2056-positive cells (mean ± SD, *n* = 3 biologically independent experiments). **e** Quantification of **d**. The percentage of pDNA-PKcs-S2056-positive cells from three independent experiments were shown in a bar chart (mean ± SD, *n* = 3 biologically independent experiments). Two-tailed unpaired *t* test with Welch’s correction was used for statistical analysis. **f** A flow cytometry analysis of pDNA-PKcs-S2056 intensity in WT, TDP1-KO, MUS81-KO, and MUS81/TDP1-DKO cells treated with 10 µM CPT for 1 h. **g** Quantification of **f**. Mean pDNA-PKcs-S2056 intensity from three independent experiments is shown in a bar chart (mean ± SD, *n* = 3). Two-tailed unpaired *t* test with Welch’s correction was used for statistical analysis. **h** A neutral comet assay was performed after treating the indicated cells with 1 µM CPT for 1 h. Olive tail movement was measured by open comet software and plotted as a box plot. The center line indicates the median, the box bounds indicate first and third quartiles, and the whiskers indicate the maximum and minimum. Numbers (No.) of cells examined were indicated. A one-way ANOVA Kruskal–Wallis test was used for statistical analysis.

It has been reported that MUS81-mediated DNA cleavage resolves replication forks stalled by TOP1 poison by generating DSBs^29^. We determined whether MUS81 is also responsible for the excess DSB generation in TDP1-KO cells. Co-depletion of MUS81 and TDP1 reduced the hyper-activation of pDNA-PKcs-S2056, pKAP1-S824, and pH2AX-S319 signal to a greater degree than did depletion of TDP1 alone (Fig. 7a). Knocking down MUS81 using siRNA also caused a reduction of the pDNA-PKcs-S2056, pKAP1-S824, and pH2AX-S319 signal in HeLa TDP1-KO cells (Supplementary Fig. 8a). MUS81 forms different complexes with EME1 or EME2^55^. When knocking down EME1 or EME2, we also observed a slight reduction of the pDNA-PKcs-S2056, pKAP1-S824, and pH2AX-S319 signal, but less than the full depletion of MUS81 (Fig. 7b, c), which suggested that both MUS81-EME1 and the MUS81-EME2 complex are involved in the generation of excess DSBs in TDP1-KO cells.

Because this function of MUS81 in generating DSBs after CPT treatment was also reported in WT cells^29^, we specifically determined the excess DSBs formed in TDP1-KO cells by monitoring pDNA-PKcs-S2056 signal using fluorescence-activated cell sorting analyses. The percentage of pDNA-PKcs-S2056-positive cells (Fig. 7d, e) and the mean pDNA-PKcs-S2056 intensity (Fig. 7f, g) was reduced with co-depletion of MUS81 and TDP1 compared to depletion of TDP1 alone. The percentage of pDNA-PKcs-S2056-positive cells did not change with depletion of MUS81 alone in WT cells, but the mean pDNA-PKcs-S2056 intensity did decrease slightly compared to in WT cells, which is consistent with the results of a previous report^29^. The cell cycle distribution did not change as a result of TDP1 or MUS81 deficiency, with or without CPT treatment (Supplementary Fig. 8b). When independently comparing the pDNA-PKcs-S2056 signal in different cell cycle phases, we found that depletion of MUS81 caused a reduction of both the percentage of positive cells and the mean intensity of pDNA-PKcs-S2056 signal in all cell cycle phases (Supplementary Fig. 8c–e). This is consistent with our above finding that TOP1cc-converted DSBs were probably generated from all cell cycle phases. In a previous report, MUS81 was only found to be associated significantly with DSBs formed in replicating cells but not in non-replicating cells^29^. One explanation for this is that the CPT concentration used here was higher and induced a large amount of DNA damage in non-replicating cells, especially in TDP1-KO cells.

We directly detected the DSB formation by neutral comet assay. Consistent with the observed reduction of the DSB-induced DDR signal, the depletion of MUS81 resulted in fewer DSBs than WT cells upon CPT treatment, and co-depletion of MUS81 and TDP1 decreased the excess DSBs that formed in TDP1-KO cells (Fig. 7h). Taken together, we suspect that MUS81 is a major factor that converts TOP1cc into DSBs after CPT treatment. We then determined whether MUS81 can directly excise the accumulated TOP1cc. A RADAR assay did not show a significant change in TOP1cc accumulation due to MUS81 deficiency in both WT and TDP1-KO cells (Supplementary Fig. 8f), which suggested that MUS81 does not function to excise TOP1cc directly; this is consistent with the results of a previous report^29^.

### Co-inhibition of TDP1 and the DSB repair pathway enhanced cellular sensitivity to CPT treatment

**Fig. 8:**
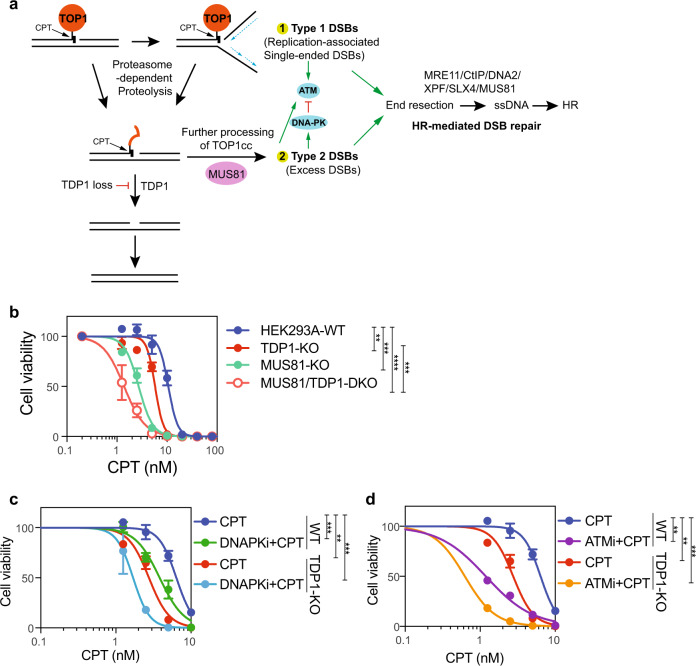
Co-inhibition of TDP1 and DSB repair pathway enhanced cellular sensitivity to CPT treatment. **a** Working model of TOP1-induced damage repair in WT and TDP1-KO cells. **b** The proliferation of HEK293A-WT, TDP1-KO, MUS81-KO, and MUS81/TDP1-DKO cells was measured using a CellTiter-Glo assay after 3 days in the presence of the indicated concentrations of CPT. Data are presented as the mean ± SD (*n* = 3 biologically independent experiments). A two-tailed unpaired *t*-test was used for statistical analysis of the IC50 of each cell line. **p (HEK293A-WT vs. TDP1-KO) = 0.005347, ***p (HEK293A-WT vs. MUS81-KO) = 0.000216, ****p (HEK293A-WT vs. MUS81/TDP1-DKO) = 0.000024, and ***p (TDP1-KO vs. MUS81-KO) = 0.000442. **c** The proliferation of HEK293A-WT and TDP1-KO cells was measured using a CellTiter-Glo assay after 7 days in the presence of the indicated concentrations of CPT and DNAPKi (AZD7648) + CPT. Data are presented as the mean ± SD (*n* = 3 biologically independent experiments). A two-tailed unpaired *t*-test was used for statistical analysis of the IC50 of each cell line. ***p (WT_CPT vs. WT_DNAPKi+CPT) = 0.000276, **p (WT_CPT vs. TDP1-KO_CPT) = 0.001041, and ***p (WT_CPT vs. TDP1-KO_DNAPKi + CPT) = 0.000685. **d** The proliferation of HEK293A-WT and TDP1-KO cells was measured using a CellTiter-Glo assay after 7 days in the presence of the indicated concentrations of CPT and ATMi (KU55933) + CPT. Data are presented as the mean ± SD (*n* = 3 biologically independent experiments). A two-tailed unpaired *t*-test was used for statistical analysis of the IC50 of each cell line. **p (WT_CPT vs. WT_ATMi+CPT) = 0.001008, **p (WT_CPT vs. TDP1-KO_CPT) = 0.001041, and ***p (WT_CPT vs. TDP1-KO_ATMi+CPT) = 0.000859.

We have demonstrated that MUS81 is required for the conversion of TOP1cc into DSBs after CPT treatment. This function was enhanced by accumulated TOP1cc in TDP1-KO cells (Fig. 8a). The original SSBs induced by TOP1 poison can be converted into single-ended DSBs by replication run-off, which activate ATM. Proteolysis of the covalently bound TOP1 made the TOP1cc-conjugated SSB ends accessible to other repair factors. The residual covalently cross-linked DNA-peptide can be quickly removed by TDP1 or APEX2 and be re-ligated. However, in TDP1-KO cells, the accumulated TOP1cc promotes replication fork reversal or R-loop formation, which can be processed by MUS81 to generate DSBs. These DSBs activate DNA-PK and ATM and were further repaired by the HR pathway.

The excess DSBs generated in TDP1-KO cells promoted HR-mediated repair of TOP1 induced damage, which somehow compensates the defects with TDP1 loss. On the basis of this finding, we suspect that co-inhibition of TDP1 and the DSB repair pathway could enhance the cellular response to TOP1-induced damage. We evaluated the cellular sensitivity of MUS81-KO and MUS81/TDP1-DKO cells to CPT treatment. MUS81-KO cells showed relatively higher sensitivity to CPT than did TDP1-KO cells, and MUS81/TDP1-DKO cells displayed an additive effect in response to CPT treatment (Fig. 8b). In WT cells, conversion of TOP1cc into DSBs should be limited because of the high efficiency of TDP1 in resolving TOP1cc. The hypersensitivity of MUS81-KO cells to CPT suggests that MUS81 has other functions besides the conversion of TOP1cc into DSBs. As a matter of fact, MUS81 has the ability to resolve holiday-junction structures during HR repair^29,56,57^, which may account for its important role in WT cells after CPT treatment. Inhibition of the DSB-induced DDR response by DNA-PK inhibitor or ATM inhibitor also showed a combinational effect with CPT treatment and had more additive effects with co-depletion of TDP1 in the cells (Fig. 8c, d).

## Discussion

In this study, we investigated TOP1-induced DNA damage repair, with or without the function of TDP1. We found more DSBs and hyper-activated DSB-induced DDR signaling in TDP1-KO cells after the induction of TOP1-induced damage, which are likely to be repair intermediates of alternative repair pathways in cells with TDP1 loss. The results of our subsequent experiments suggest that MUS81 is responsible for the formation of these DSBs, especially in TDP1-KO cells, indicating that MUS81 is the key player involved in the processing and repair of TOP1-induced DNA damage. These increased DSBs are accompanied by enhanced end resection, which we showed is dependent on several repair factors that are known to be involved in HR repair, including MRE11, CtIP, DNA2, XPF, and probably also MUS81 (Figs. 6a–f, 7a–c). Thus, our current hypothesis is that TOP1cc can be either resolved directly by TDP1/APEX2 or converted into DSBs and repaired further by the HR pathway (Fig. 8a). In proliferating cells, high HR activity may somehow substitute for TDP1 function and alleviate the cellular sensitivity of TDP1-KO cells to CPT treatment. However, the conversion and repair of DSBs may not be efficient in non-dividing cells. This may explain why TDP1 deficiency does not cause obvious defects in rapidly replicating tissues, but instead causes spinocerebellar ataxia with axonal neuropathy by affecting terminally differentiated, non-dividing neuronal cells^12^.

CPT-induced TOP1cc can be rapidly converted into DNA lesions by DNA and RNA synthesis^4,30,58^, probably by replication run-off at the original single-strand breaks (SSBs) induced by TOP1 poison, and therefore form single-ended DSBs^4,28^. ATM may be directly activated by such replication-associated single-ended DSBs induced by CPT treatment, which are not preferable substrates for DNA-PKcs. Previous reports suggested that ATM even releases both KU and DNA-PKcs from single-ended DSBs^59,60^. Here, we found that ATM was phosphorylated quickly after CPT treatment in both WT and TDP1-KO cells (Fig. 1e and Supplementary Fig. 2a). On the other hand, the phosphorylation of DNA-PK was delayed and only became obvious 60 min (60’) or more after CPT treatment in both WT and TDP1-KO cells (Fig. 1e and Supplementary Fig. 2a), which suggests that a different type of DSBs formed after the initial formation of single-ended DSBs when replication forks encountered TOP1cc after CPT treatment (Fig. 2b). The notable hyper-phosphorylation of DNA-PKcs in TDP1-KO cells indicated the enhanced formation of type 2 DSB formation, which might be generated from inefficiently repaired proteolyzed TOP1cc.

MUS81 is a DNA structure–specific endonuclease that can cut on reversed replication forks, 3’ flap structures, holiday junctions, and D-loops^29,56,57^. The involvement of MUS81 in generating TOP1-induced DSBs has been suggested^29,61^ and was supposed to cleave on the replication forks that stalled by TOP1cc. Moreover, it has been shown that TOP1 poison results in PARP-mediated replication fork reversal^62^, which is also a substrate that can be processed by MUS81. Here, we found that besides S phase cells, MUS81 is also responsible for TOP1-induced DSB formation in non-S phase cells. CPT treatment can stall transcription elongation and induce R-loop formation^27,61^. The formation of transcription-dependent TOP1-induced DSBs has been shown to be dependent on R-loops^27^. The function of MUS81 in preventing R loop-induced genome instability has been suggested in replicating S phase cells^63,64^. Our finding suggests a role for MUS81 in generating transcription-coupled TOP1-induced DSBs in non-S phase cells, probably by cleavage of R-loops. How MUS81 cleaves on the stalled transcription-associated DNA structure still needs further illustration. On the other hand, the nuclease activity of MUS81 is cell cycle-regulated^55^, indicating that there are distinct mechanisms underlying the generation of TOP1-induced DSBs by MUS81 in different cell cycle phases.

TDP1 is a specific tyrosyl-DNA phosphodiesterase that cells have evolved to resolve the covalent bond between TOP1 catalytic tyrosine and the 3’ end of DNA^9,10^. Using unbiased whole genome screens, we identified the synthetic lethality between APEX1/APEX2 with TDP1, which has also been indicated before^41^. We then proved that the co-lethality between APEX1 and TDP1 is due to their redundant function in repairing base damage. Co-depletion of TDP1 and APEX1 reduced the cell viability graduality, indicating an accumulation of unrepaired endogenous DNA damage during proliferation. On the other hand, APEX2 had an additive effect with TDP1 in both CPT and MMS treatment (Fig. 5g, h), suggesting that APEX2 and TDP1 have redundant roles in both base damage repair and TOP1-induced DNA damage repair. The function of APEX2 was further proved by the accumulation of TOP1cc with APEX2 deficiency in WT cells, which agreed with the observation that APEX2 processed the TOP1cc mimicking structure in vitro^41^. However, we failed to observe any additive effect on TOP1cc accumulation with TDP1 and APEX2 co-deficiency. We cannot exclude the possibility that APEX2 promotes TOP1cc removal with TDP1 loss. It would be interesting to determine how TDP1 and APEX2 are coordinated and regulated in the resolution of TOP1cc in vivo.

In summary, our study reveals the TDP1-dependent and -independent pathways that are involved in the signaling, processing, and repair of TOP1-induced damage. It is likely that our mechanistic investigation of these pathways will contribute to the development of combinational TOP1-inhibitor–based cancer therapies.

## Methods

### Cell culture

HEK293A cells were purchased from ThermoFisher (R70507). HeLa, and HEK293T cells were purchased from the ATCC (Manassas, VA). All cell lines were maintained in Dulbecco’s modified Eagle’s medium containing 10% fetal calf serum at 37 °C with 5% CO_2_.

### Antibodies, chemicals, siRNAs, and sgRNAs

In this study, the antibodies used for Western blotting included TOP1cc (Clone 1.1 A, MABE1084; Millipore; 1: 1000 dilution), dsDNA (ab27156; Abcam; 1: 5000 dilution), phospho-DNA-PKcs (S2056, ab18192; Abcam; 1: 1000 dilution), phospho-KAP1 (S824, 4127 S; Cell Signaling Technology; 1: 2000 dilution), phospho-ATM (S1981, ab81292; Abcam; 1: 1000 dilution), phospho-ATM (S1981, 13050 S; Cell Signaling Technology; 1: 1000 dilution), phospho-Chk2 (Thr68, 2661 S; Cell Signaling Technology; 1: 1000 dilution), phospho-H2AX (S139, 9718 S; Cell Signaling Technology; 1: 1000 dilution), phospho-H2AX (S139, 05-636 l; Millipore; 1: 1000 dilution), phospho-Chk1 (Ser345, 2348 S; Cell Signaling Technology; 1: 1000 dilution), phospho-Chk1 (Ser317, 12302 S; Cell Signaling Technology; 1: 1000 dilution), phospho-RPA32 (S4/S8, A300-245A; Bethyl Laboratories; 1: 1000 dilution), Chk2 (6334 S; Cell Signaling Technology; 1: 1000 dilution), KAP1 (A300-274A; Bethyl Laboratories; 1: 1000 dilution), TDP1 (sc-365674; Santa Cruz Biotechnology; 1: 500 dilution), TDP2 (sc-377280; Santa Cruz Biotechnology; 1: 500 dilution), APEX1 (4128 S; Cell Signaling Technology; 1: 1000 dilution), XPF (A301-315A; Bethyl Laboratories; 1: 1000 dilution), MUS81 (sc-53382; Santa Cruz Biotechnology; 1: 500 dilution), SLX4 (in-house developed antibody against the antigen comprising SLX4^231–460^; 1: 1000 dilution), EME1 (ab88878; Abcam; 1: 1000 dilution), EME2 (NBP3-04534-100UL; Novus Biologicals; 1: 1000 dilution), MRE11 (NB100-142; Novus Biologicals; 1: 1000 dilution), CtIP (9201 S; Cell Signaling Technology; 1: 1000 dilution), DNA2 (ab96488, Abcam; 1: 1000 dilution), H3 (ab1791; Abcam; 1: 2000 dilution), Actin (A5441-100UL; Sigma-Aldrich; 1: 4000 dilution), Vinculin (V9131; Sigma-Aldrich; 1: 4000 dilution), Tubulin (T6199-200UL; Sigma-Aldrich; 1: 4000 dilution), goat anti-rabbit IgG (H + L) cross-adsorbed secondary antibody, FITC (F-2765; Thermo Fisher Scientific; 1: 1000 dilution), goat anti-mouse IgG (H + L) highly cross-adsorbed secondary antibody, and Alexa Fluor™ Plus 488 (A32723, Thermo Fisher Scientific; 1: 1000 dilution).

The chemicals used in this study included AZD6738 (ATR kinase inhibitor, S7693; Selleck Chemicals), AZD0156 (ATM kinase inhibitor, S8375; Selleck Chemicals), KU-55933 (ATM kinase inhibitor, S1092; Selleck Chemicals), AZD7648 (DNA-PK inhibitor, S8843; Selleck Chemicals), MG132 (S2619; Selleck Chemicals), APE1 redox inhibitor E3330 (E8534-5MG; Sigma-Aldrich), APE1 inhibitor III (262017-10MG; Millipore Sigma), CPT (390238-25MG; Calbiochem), etoposide (ETO, E1383100MG; Fisher Scientific), aphidicolin (APH, A0781-5MG; Sigma-Aldrich), 5,6-dichlorobenzimidazole 1-B-D-ribofuranoside (DRB, D1916-10MG; Sigma-Aldrich), and methyl methanesulfonate (MMS, AAH5512006; Fisher Scientific).

ON-TARGETplus human siRNA smart pool for MRE11 (L-009271-00-0005), EME1 (L-016420-01-0005), EME2 (L-032783-02-0005), DNA2 (L-026431-01-0005), SLX4 (L-014895-00-0005), KAP1 (L-005046-00-0005), and ON-TARGETplus non-targeting siRNA (D-001810-01-20) were purchased from Dharmacon. MUS81 Human siRNA Oligo Duplex (SR312835) was purchased from Origene. siRNAs were transfected into cells using Lipofectamine™ RNAiMAX Transfection Reagent (13778075; Thermo Fisher Scientific).

### CRISPR/Cas9-mediated gene knockout

The knockout cell lines used in this study were generated as previously described^48^. In brief, sgRNAs targeting a specific gene were ligated into pLenti-V2 plasmid^65^. The pLenti-V2-sgRNA plasmids were then transfected into cells using polyethylenimine. After transfection, cells were selected with puromycin for 2 days and then diluted and seeded into 96-well plates. Ten days after seeding, single clones were selected and subjected to Western blotting to determine the expression of the targeted gene or protein.

For knockdown of gene expression with sgRNAs in some experiments, the pLenti-V2 empty plasmid or pLenti-V2-sgRNA was packed into lenti-virus. Targeted cell lines were infected with either pLenti-V2 empty virus or pLenti-V2-sgRNA virus. Cells were then selected with puromycin for two rounds, with each round lasting 2 days. Pooled cells were used for further experiments.

XPF-KO and MUS81-KO cells have been described previously^48^. The gRNAs used in this study were (1) TDP1: AAGGAGCAGCAAATGAGCCC, (2) TDP2: TCTCCCAGTCGTTCTCGGCC, (3) APEX1-1: GATCAGAAAACCTCACCCAG, (4) APEX1-2: AGGACAGTGATCACTGCCGA, and (5) APEX2: AGATGTTGCGCGTGGTGAGC.

### Slot blot for TOP1cc detection

To purify TOP1cc from the cells, we used the RADAR (rapid approach to DNA adduct recovery) assay, as described previously^19^. In brief, cells were plated in six-well plates and treated with 10 μM CPT for 1 h. When collecting samples, cells were washed with 1× phosphate-buffered saline (PBS) and directly lysed by adding 1 mL of MB buffer (6 M guanidinium isothiocyanate, 10 mM Tris-HCl [pH 6.8], 20 mM EDTA, 4% Triton X-100, 1% sarkosyl, and 1% dithiothreitol) into the cell culture plates. DNA was precipitated by adding 0.5 mL of 100% ethanol and washed three times with 70% ethanol. DNA pellets were then resolved in 200 μL of 8 mM NaOH. A small fraction of DNA was treated with RNAase A at 37 °C for 1 h and quantified using a NanoDrop spectrophotometer (Thermo Scientific). Samples were diluted and applied to a Nitrocellulose (Millipore) membrane using a vacuum slot-blot manifold, with 3 μg of DNA loaded for each sample. Staining with dsDNA antibody was used as the loading control. The results were quantified with Image J software (version 1.50i). Relative TOP1cc density was calculated and normalized with loading controls. Samples of WT cells with CPT treatment were set to 1 to compare relative TOP1cc density from different repeats.

### Genome-wide CRISPR/Cas9 screens

Unbiased genomic CRISPR gRNA screening was conducted as described previously^31^ using the Toronto Knock Out Library v3 gRNA library. In brief, 120 million HEK293A wild-type (WT) and TDP1-KO cells were infected separately with the Toronto Knock Out Library v3 lentiviruses at a low MOI (<0.3). Twenty-four hours after infection, infected cells were trypsin-digested and re-cultured in fresh Dulbecco’s modified Eagle’s medium supplemented with 2 μg/mL puromycin. Two days after selection, the remaining cells were sub-divided into different groups, with three replicates and at least 20 million cells in each group. The day was set as day 0 (T0). Cells were then sub-cultured every 3 days, with or without CPT treatment, for a total of 21 days. A cell number of at least 20 million was maintained for every sub-culture cycle. Finally, 20 million cells from T0 and T21 were collected for genomic DNA extraction using a QIAamp Blood Maxi Kit (QIAGEN). gRNAs that inserted into the genome were amplified via PCR using primers harboring Illumina TruSeq adapters with i5 and i7 barcodes, as described previously^31^. The resulting PCR products were purified and sequenced using an Illumina HiSeq 2500 system. Data analyses were conducted with a model-based analysis of genome-wide CRISPR/Cas9 knockout (MAGeCK, https://sourceforge.net/p/mageck/wiki/Home/)^66^ and drug Z (https://github.com/hart-lab/drugz)^67^. CPT sensitivity profiling or TDP1 co-lethality profiling was analyzed by comparing the differences in gRNA abundance in different groups using drug Z.

### Colony formation assay

We seeded 200, 400, or 800 HEK293A-WT, TDP1-KO, APEX1-KO, and TDP1/APEX1-double-knockout cells (DKO) cells into six-well plates. Ten days after incubation, cells were washed with PBS and stained with a crystal violet solution to visualize colonies.

For the drug sensitivity assay, 200 cells were seeded for not treated (NT), 400 for 0.1 μM ETO and 10 nM CPT, and 1600 for 0.2 μM ETO and 20 nM CPT. Cells were treated with the indicated drugs, starting on the second day, for 24 h. Cells were then washed to remove the drug and incubated for 10 more days before being stained with crystal violet solution to visualize colonies. Colonies were manually counted, and data were plotted as surviving fractions relative to untreated cells.

### CellTiter-Glo assay

Diluted cells (100 μL [1000 cells for 3 days of drug treatment and 100 cells for 7 days of drug treatment]) were seeded onto 96-well plates. On the second day, 10 μL of serially diluted concentrations of CPT or other chemicals was added. Cells were then incubated for another 3 days or 7 days in the presence of CPT or other chemicals. On the day of analysis, cell culture media was removed, and 100 μL of CellTiter-Glo (G7572; Promega, Madison, WI) reagents were added to induce cell lysis. The plates were incubated at ambient temperature for 15 min in the dark. After incubation, 80 μL of cell lysates were transferred into opaque-walled, 96-well plates and subjected to luminescence detection using a BioTek Synergy™ 2 Multi-Mode Microplate Reader.

To detect cell proliferation with the CellTiter-Glo assay, we seeded 100 μL (100 cells) of diluted cells into 96-well plates. Cell growth was analyzed after 1, 4, and 7 days using CellTiter-Glo reagents.

### Whole-cell extract and chromatin extract preparation

For the preparation of whole-cell extract, the cell culture medium was removed, and the cells were washed with PBS. To lyse cells, 1× Laemmli buffer was added into cell culture plates. The cell lysate was then boiled at 95 °C for 10 min and subjected to Western blotting.

To separate soluble and chromatin fractions, cells were collected and lysed with ice-cold NETN buffer (50 mM Tris-HCl [pH 7.4], 100 mM NaCl, 0.4% NP-40, and 1 mM EDTA) supplemented with protease inhibitor cocktail. After being incubated for 10 min in a cold room, cell lysates were then centrifuged at 14,000 g for 10 min. The supernatant was considered a soluble fraction. The left pellet was washed twice with NETN buffer supplemented with 0.34 M sucrose and protease inhibitor cocktail and boiled at 95 °C for 10 min in 1× Laemmli buffer, which was considered the chromatin fraction.

### Neutral comet assay

A neutral comet assay was conducted using a CometAssay kit (4250-050-K; Trevigen) following the manufacturer’s instructions. In brief, the indicated cell lines were treated with 1 μM CPT for 1 h or left untreated. Cells were then trypsin-digested and re-suspended in PBS at a concentration of 1 × 10^5^ cells/ml. We then mixed 50 μL of cell suspension with 500 μL of pre-warmed LMAgarose and immediately spread the mixture onto CometSlide. The spread slides were placed at 4 °C for 15 min. Samples were lysed by immersing slides in lysis solution for 1 h at 4 °C. After being removed from the lysis solution, slides were washed and immersed in 1X neutral electrophoresis buffer (50 nM Tri base and 150 mM sodium acetate) for 30 min and then subjected to electrophoresis at 21 V for 45 min in 1X neutral electrophoresis buffer. Slides were placed in DNA precipitation solution (43.3 mL of 95% ethanol and 6.7 mL of 7.5 M ammonium acetate) for 30 min and 70% ethanol for 30 min at ambient temperature. Slides were dried overnight and stained with SYBR-gold. Images were obtained using a Nikon 90i microscope at ×20 magnification. Collected images were analyzed using OpenComet (v1.3.1 from https://cometbio.org), and the olive tail movements are shown.

### Fluorescence-activated cell sorting analyses

Cells were pre-treated with drugs, as shown in each figure. After treatment, they were collected and fixed with ice-cold 70% ethanol at 4 °C overnight. Cells were then washed with phosphate-buffered saline (PBS) and permeabilized with 0.5% Triton X-100/PBS at ambient temperature for 10 min. After being washed with PBS, cells were blocked with 4% bovine serum albumin (BSA)/PBS for 1 h and incubated with phospho-DNA-PKcs (S2056, ab18192; Abcam) antibody diluted in 4% BSA/PBS for 1 h at ambient temperature. Cells were washed with PBS three times and incubated with fluorescently labeled secondary antibodies diluted in 4% BSA/PBS for 1 h. Cells were stained with propidium iodide (PI; 20 μg/ml) and RNase A (10 μg/ml) before analysis. Data were collected with a BD C6 flow cytometer (Becton Dickinson) or Attune Flow cytometers (ThermoFisher) and analyzed with FlowJo software (FlowJo 10.6.1, Becton Dickinson).

### Two-color competitive growth assay

WT or TDP1-KO cells were infected with virus particles expressing plenti-V2-RFP-sgAAVS1 (control) or a plenti-V2-GFP-sgRNA targeting APEX1 or APEX2. Seventy-two hours after infection, RFP- and GFP-expressing cells were mixed 1:3. A fraction of mixed cells were seeded into plates, and the remaining mixed cells were analyzed by Attune Flow cytometers (ThermoFisher). During the course of the experiments, cells were sub-cultured at the indicated time points and analyzed by Attune Flow cytometers (ThermoFisher). The day of the mixture was set as day 0. Collected data were analyzed with FlowJo software (FlowJo 10.6.1, Becton Dickinson), and the relative ratios of GFP- and RFP-positive cells were calculated.

### Statistical analysis

A statistical analysis was performed using GraphPad Prism 8.0.0. A two-tailed unpaired t test with Welch’s correction was used to perform a statistical analysis of the comparison of two samples. The one-way ANOVA Kruskal–Wallis test was used to perform a statistical analysis of multiple group comparisons. All experiments were repeated at least three times, and similar results were obtained.

### Reporting summary

Further information on research design is available in the Nature Research Reporting Summary linked to this article.

## Supplementary information

Supplementary Information

Description of Additional Supplementary Files

Supplementary Data 1

Supplementary Data 2

Supplementary Data 3

Supplementary Data 4

Supplementary Data 5

Supplementary Data 6

Supplementary Data 7

Reporting Summary

## Data Availability

The data that support this study are available from the corresponding author upon reasonable request. The genome-wide CRISPR/Cas9 screen data generated in this study are provided in the Supplementary data files. Source data are provided with this paper.

## Source data

Source Data
