# Supplementary material for: TDP1-independent pathways in the process and repair of TOP1-induced DNA damage
Source: Nat Commun. 2022 Jul 22;13:4240. doi: 10.1038/s41467-022-31801-7 (PMC9307636; doi:10.1038/s41467-022-31801-7)
Supplement: Supplementary file 1 — Supplementary Information [file 41467_2022_31801_MOESM1_ESM.pdf]

Supplementary Information

TDP1-independent pathways in the process and repair of TOP1-induced DNA damage

Huimin Zhang[1#], Yun Xiong[1#], Dan Su[1], Chao Wang[1], Mrinal Srivastava[1], Mengfan Tang[1], Xu Feng[1], Min Huang[1], Zhen Chen[1], Junjie Chen[1*]

Affiliations

[1]Department of Experimental Radiation Oncology, The University of Texas MD Anderson Cancer Center, Houston, TX 77030, USA

[#]These authors contributed equally: Huimin Zhang, Yun Xiong.

*Lead contact and corresponding author. Please address correspondence to jchen8@mdanderson.org

Supplementary Fig. 1

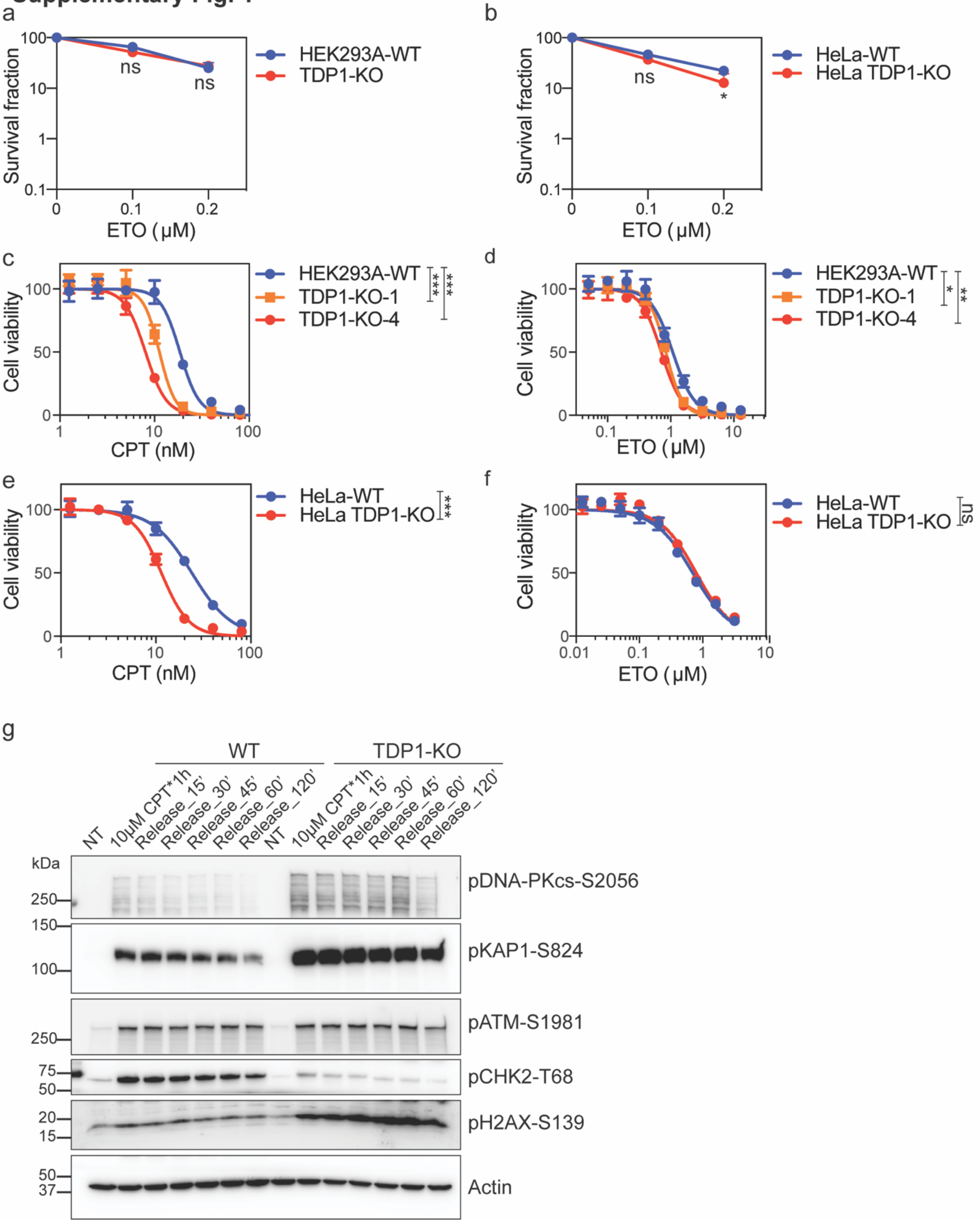

Supplementary Fig. 1: TDP1-KO cells showed cellular sensitivity to TOP1 poison camptothecin (CPT). a Colony formation assay of HEK293A-WT and TDP1-KO cells with ETO treatment. Data are represented as mean ± SD. n=6 biologically independent replicates. Two-tailed unpaired *t* test with Welch's correction was used for statistical analysis. ns, not significant. **b** Colony formation assay of HeLa-WT and HeLa TDP1-KO cells with ETO treatment. Data are represented as mean ± SD. n = 6 biologically independent replicates. Two-tailed unpaired *t* test with Welch's correction was used for statistical analysis. *p = 0.0179, and ns, not significant. **c** The proliferation of HEK293A-WT, TDP1-KO-1, and TDP1-KO-4 cells was measured using a CellTiter-Glo assay after 3 days in the presence of the indicated concentrations of CPT. Data are presented as the mean ± SD (n = 3 biologically independent replicates). Two-tailed unpaired *t*-test was used for statistical analysis of the IC50 of each cell line. ***p (HEK293A-WT vs. TDP1-KO-1) = 0.00029, and ***p (HEK293A-WT vs. TDP1-KO-4) = 0.00029. **d** as in **c** but treated with different concentrations of ETO. Data are presented as the mean ± SD (n = 3 biologically independent replicates). Two-tailed unpaired *t*-test was used for statistical analysis. *p (HEK293A-WT vs. TDP1-KO-1) = 0.01418, and **p (HEK293A-WT vs. TDP1-KO-4) = 0.00363. **e** as in **c** but the proliferation of HeLa and HeLa TDP1-KO cells was measured. Data are presented as the mean ± SD (n = 3 biologically independent replicates). Two-tailed unpaired *t*-test was used for statistical analysis. ***p = 0.00031. **f** as in **c** but the proliferation of HeLa and HeLa TDP1-KO cells with ETO treatment was measured. Data are presented as the mean ± SD (n = 3 biologically independent replicates). Two-tailed unpaired *t*-test was used for statistical analysis. ns, not significant. **g** WT and TDP1-KO cells were treated with 10 μM CPT for 1 h and then

released into fresh medium and cultured for the indicated times. Whole-cell extracts were prepared and subjected to Western blotting with the indicated antibodies. Experiments were repeated at least three times, and similar results were obtained.

Supplementary Fig. 2

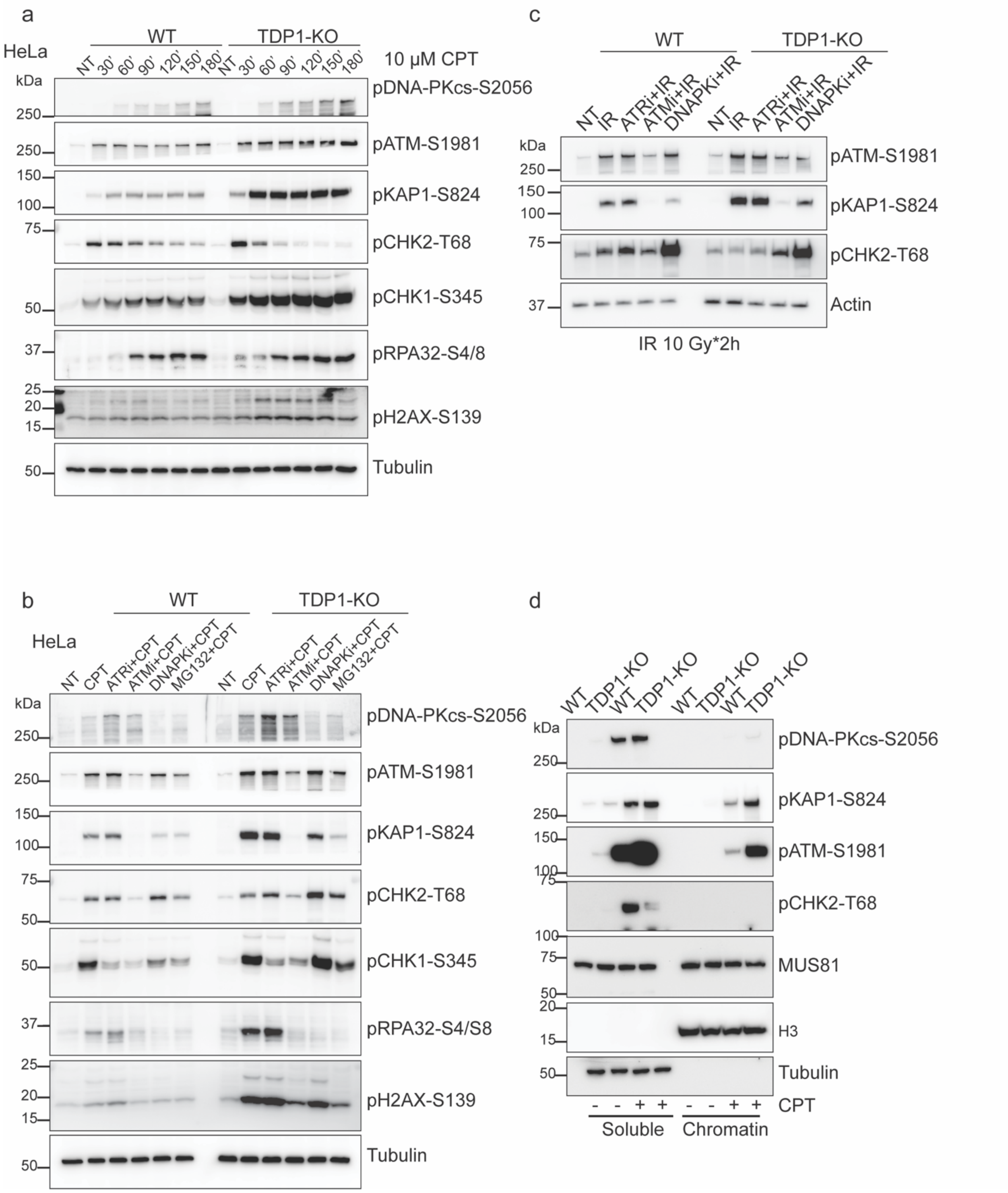

Supplementary Fig. 2: TDP1-KO cells show increased DSB-induced DNA damage response after treatment with TOP1 poison in HeLa cells. a HeLa WT and HeLa TDP1-KO cells were treated with 10 μM CPT for the indicated times. Whole-cell extracts were prepared and subjected to Western blotting with the indicated antibodies. Experiments were repeated at least three times, and similar results were obtained. **b** HeLa WT and HeLa TDP1-KO cells were pre-treated with 10 μM ATRi (AZD6738), 1 μM ATMi (AZD0156), 10 μM DNA-PKi (AZD7648), or 10 μM MG132 for 1 h and then treated with 10 μM CPT for 1 h. Whole-cell extracts were prepared and subjected to Western blotting with the indicated antibodies. Experiments were repeated at least three times, and similar results were obtained. **c** WT and TDP1-KO cells were pre-treated with 10 μM ATRi (AZD6738), 10 μM ATMi (KU55933), or 10 μM DNA-PKi (AZD7648) and subjected to 10 Gy IR and then released for 2 h. Whole-cell extracts were prepared and subjected to Western blotting with the indicated antibodies. Experiments were repeated at least three times, and similar results were obtained. **d** WT and TDP1-KO cells were untreated (NT) or treated with 10 μM CPT for 1 h. The soluble fraction and chromatin fraction were separated and subjected to Western blotting with the indicated antibodies. Experiments were repeated at least three times, and similar results were obtained.

Supplementary Fig. 3

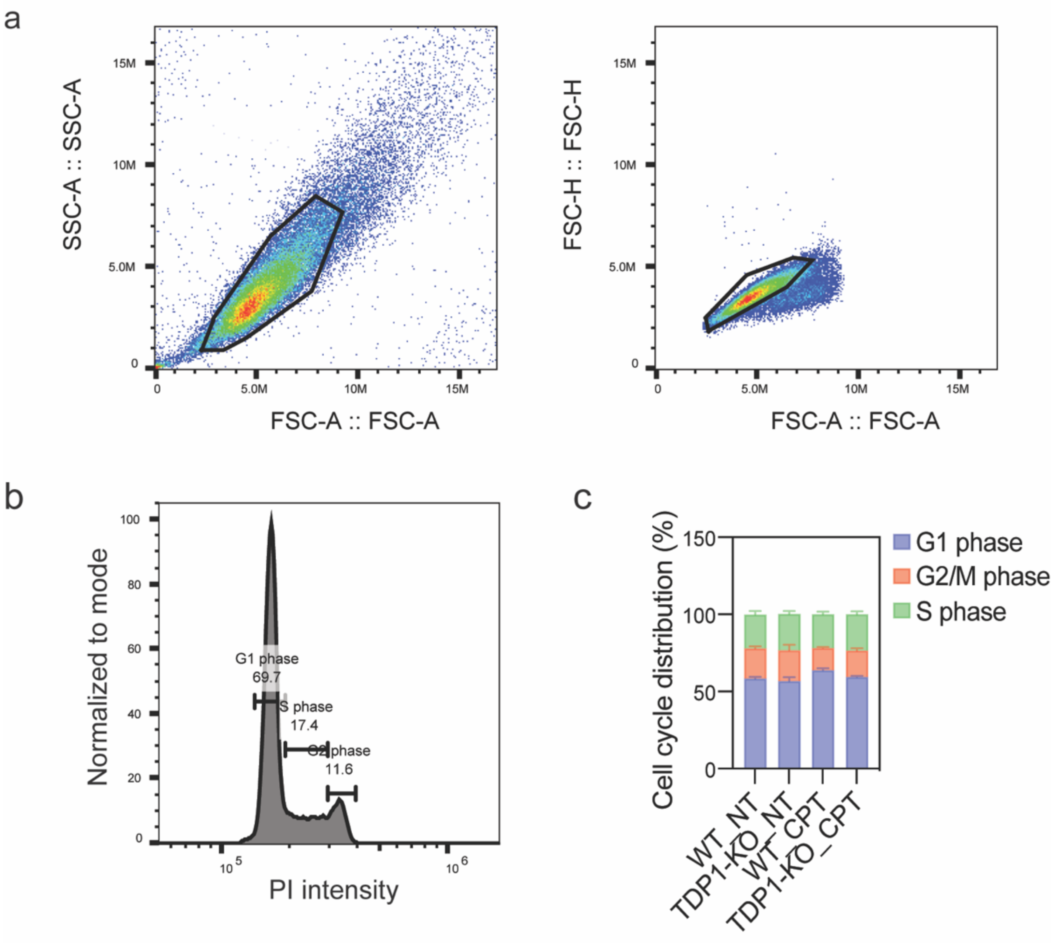

Supplementary Fig. 3: Example of flow cytometry gating strategy. a Example of flow cytometry gating strategy for single cells. **b** Example of flow cytometry gating strategy for G1, S, and G2/M phases. **c** Cell cycle distribution of the flow cytometry analysis of WT and TDP1-KO cells either not treated (NT) or treated with 10 μM CPT for 1 h. Data are presented as the mean ± SD (n = 3 biologically independent experiments).

Supplementary Fig. 4

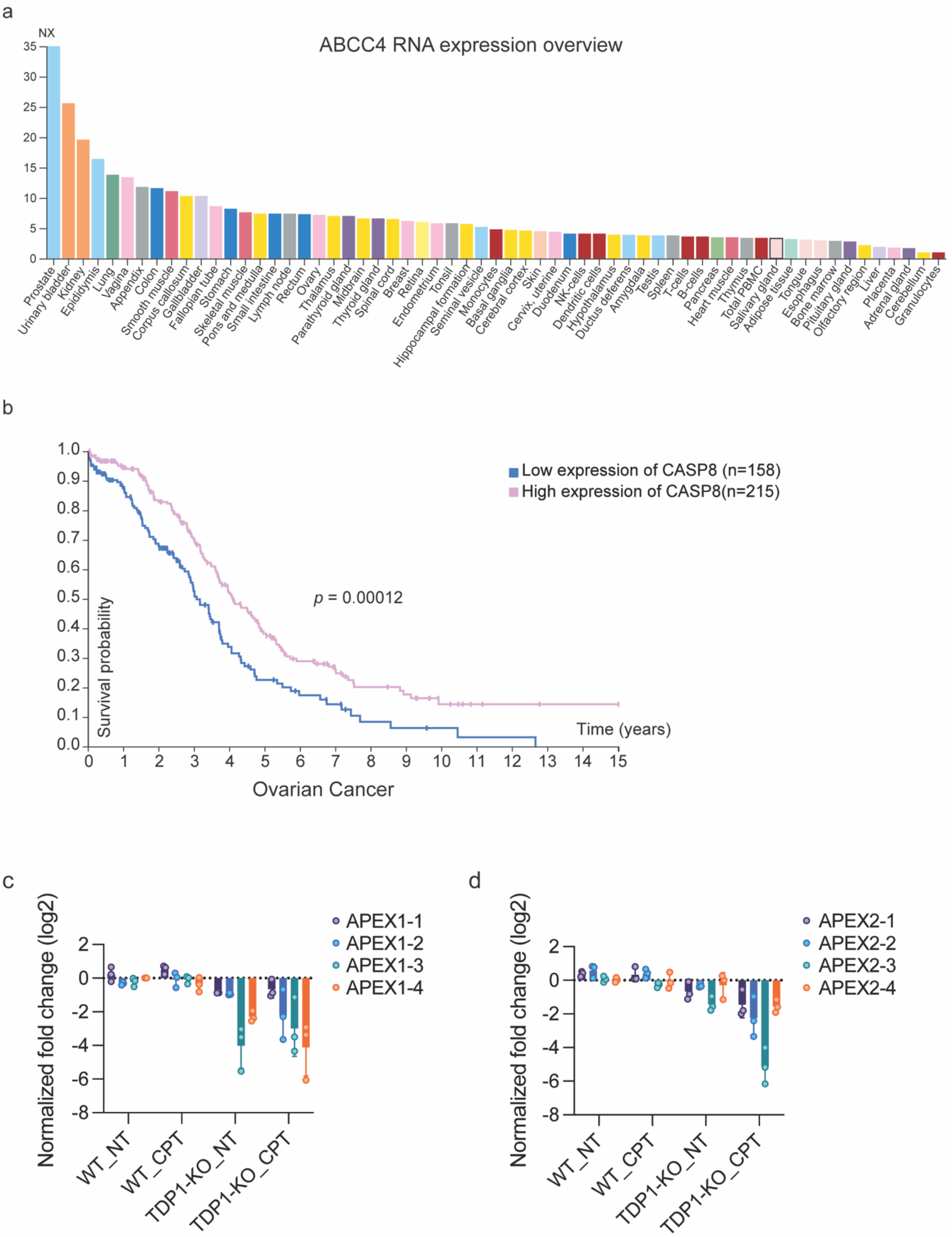

Supplementary Fig. 4: Genome-wide CRISPR/Cas9 screens in wild-type and TDP1-KO cells with TOP1 poison. a ABCC4 RNA expression overview. Data were obtained from The Human Protein Atlas database (https://www.proteinatlas.org/). **b** Kaplan-Meier plots for ovarian cancer, in which high expression of CASP8 is significantly associated with survival (p = 0.00012). Data were obtained from The Human Protein Atlas database (https://www.proteinatlas.org/). p score is the log rank p value for Kaplan-Meier plot showing results from analysis of correlation between mRNA expression level and patient survival. **c** Normalized fold-changes of sgRNA reads for APEX1 in WT_NT, WT_CPT, TDP1-KO_NT, and TDP1-KO_CPT groups. Data are presented as the mean ± SD (n = 3 biologically independent experiments). **d** Normalized fold-changes of sgRNA reads for APEX2 in WT_NT, WT_CPT, TDP1-KO_NT, and TDP1-KO_CPT groups. Data are presented as the mean ± SD (n = 3 biologically independent experiments).

Supplementary Fig. 5

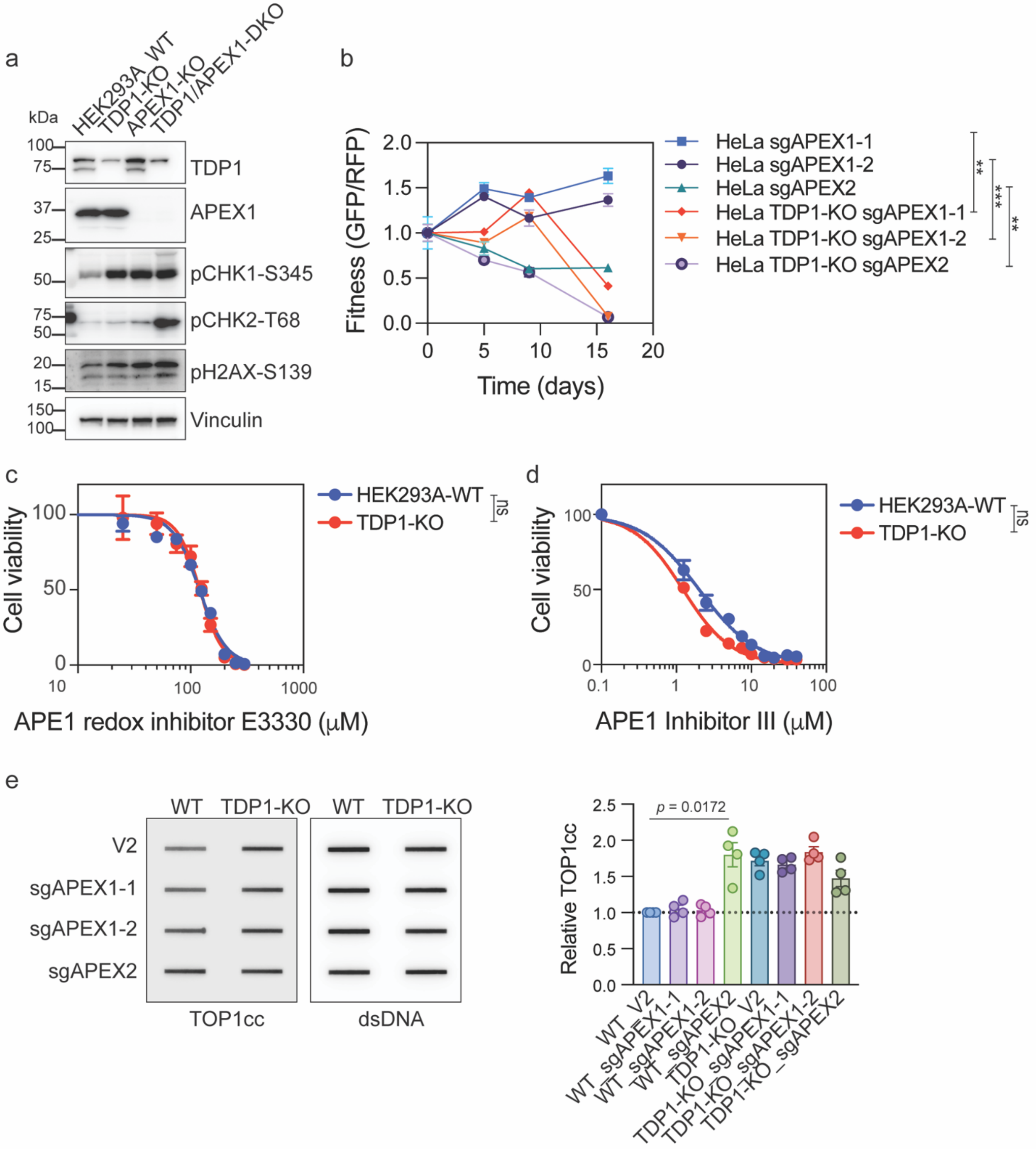

Supplementary Fig. 5: APEX1 and APEX2 are synthetic lethal with TDP1. a DNA damage checkpoint signaling was detected in HEK293A-WT, TDP1-KO, APEX1-KO, and TDP1/APEX1-DKO cells. Whole-cell extracts were prepared and subjected to Western blotting with the indicated antibodies. Experiments were repeated at least three times, and similar results were obtained. **b** Competitive growth assays in HeLa WT and HeLa TDP1-KO cells infected with a virus expressing the indicated sgRNAs. Data are presented as mean ± SD (n = 3 biologically independent experiments). Two-tailed unpaired *t* test with Welch's correction was used for statistical analysis. ***p* (HeLa sgAPEX1-1 vs. HeLa TDP1-KO sgAPEX1-1) = 0.001301, ****p* (HeLa sgAPEX1-2 vs. HeLa TDP1-KO sgAPEX1-2) = 0.000939, and ***p* (HeLa sgAPEX2 vs. HeLa TDP1-KO sgAPEX2) = 0.001705. **c, d** Proliferation of HEK293A-WT and TDP1-KO cells was measured using a CellTiter-Glo assay after 3 days in the presence of the indicated concentrations of APEX1 redox inhibitor E3330 (**c**) or APEX1 inhibitor III (**d**). Data are presented as the mean ± SD (n = 3 biologically independent experiments). A two-tailed unpaired *t*-test was used for statistical analysis of the IC50 of each cell line. ns, not significant. **e** WT and TDP1-KO cells were infected with pLenti-V2 empty vector or pLenti-V2-sgRNAs targeting APEX1 or APEX2. A RADAR assay was used to assess TOP1cc accumulation after cells were treated with 10 μM CPT for 1 h. Double-stranded DNA (dsDNA) was used as a loading control. Data are represented as mean ± SEM (n = 4 biologically independent experiments). Two-tailed unpaired *t* test with Welch's correction was used for statistical analysis.

Supplementary Fig. 6

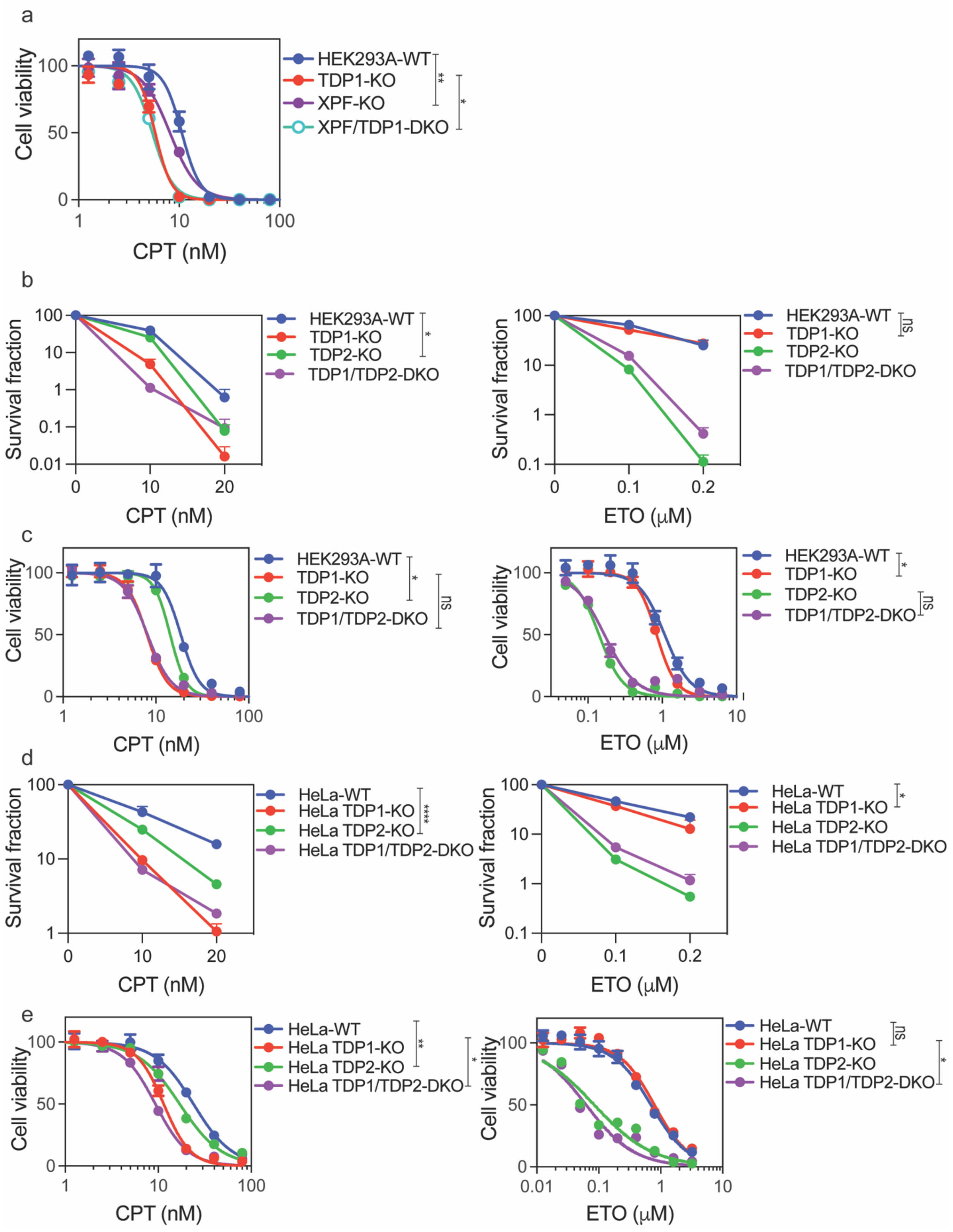

Supplementary Fig. 6: TDP2-KO cells showed mild cellular sensitivity to CPT and no additive effect with TDP1 deficiency. a The proliferation of indicated cell lines was measured using a CellTiter-Glo assay after 3 days in the presence of the indicated concentrations of CPT. Data are presented as the mean ± SD (n = 3 biologically independent experiments). Two-tailed unpaired *t*-test was used for statistical analysis of the IC50 of each cell line. ***p* (HEK293A-WT vs. XPF-KO) = 0.003893, and **p* (TDP1-KO vs. XPF/TDP1-DKO) = 0.044978. **b** CPT (left panel) and ETO (right panel) sensitivity of indicated cell lines was detected by colony formation assay. Data are represented as mean ± SD. n = 6 biologically independent replicates. Two-tailed unpaired *t* test with Welch's correction was used for statistical analysis of 20 nM CPT or 0.2 μM ETO. **p* (CPT: HEK293A-WT vs. TDP2-KO) = 0.02007, and ns, not significant. **c** as in **a** but the proliferation of indicated cell lines was measured in the presence of CPT (left panel) or ETO (right panel). Data are presented as the mean ± SD (n = 3 biologically independent experiments). Two-tailed unpaired *t*-test was used for statistical analysis. **p* (CPT: HEK293A-WT vs. TDP2-KO) = 0.013910, **p* (ETO: HEK293A-WT vs. TDP1-KO) = 0.014183, and ns, not significant. **d** as in **b** but indicated HeLa cell lines was examined. Data are represented as mean ± SD. n = 6 biologically independent replicates. Two-tailed unpaired *t* test with Welch's correction was used for statistical analysis. *****p* (CPT: HeLa-WT vs. HeLa TDP2-KO) = 0.000003, **p* (ETO: HeLa-WT vs. HeLa TDP1-KO) = 0.017941. **e** as in **a** but indicated HeLa cell lines was measured in the presence of CPT (left panel) or ETO (right panel). Data are presented as the mean ± SD (n = 3 biologically independent experiments). Two-tailed unpaired *t*-test was used for statistical analysis. ***p* (CPT: HeLa-WT vs. HeLa TDP2-KO) = 0.001558, **p* (CPT:

HeLa TDP1-KO vs. HeLa TDP1/TDP2-DKO) = 0.022181, *p (ETO: HeLa TDP1-KO vs. HeLa TDP1/TDP2-DKO) = 0.018112, and ns, not significant.

Supplementary Fig. 7

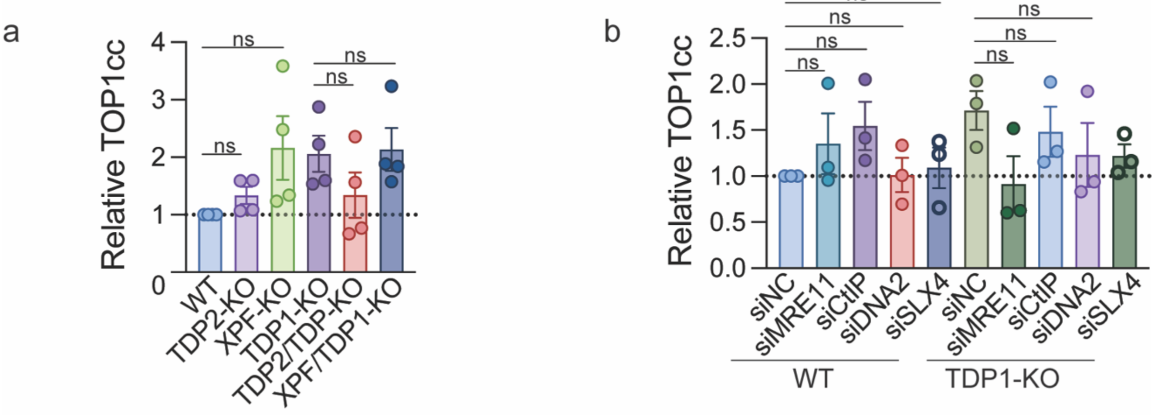

Supplementary Fig. 7: TOP1cc accumulation was monitored by RADAR assay with deficiency of XPF, TDP2, MRE11, CtIP, DNA2 and SLX4 in WT and TDP1-KO cells. a A RADAR assay was performed after treating the indicated cells with 10 μM CPT for 1 h to assess the TOP1cc accumulation. Data are represented as mean ± SEM (n = 4 biologically independent experiments). A two-tailed unpaired *t* test with Welch's correction was used for statistical analysis. ns, not significant. **b** Control siRNA or siRNA against SLX4, MRE11, CtIP, or DNA2 were transfected into WT or TDP1-KO cells. 72 h after siRNA transfection, a RADAR assay was performed after treating the indicated cells with 10 μM CPT for 1 h to assess TOP1cc accumulation. Data are represented as mean ± SEM (n = 3 biologically independent experiments). A two-tailed unpaired *t* test with Welch's correction was used for statistical analysis. ns, not significant.

Supplementary Fig. 8

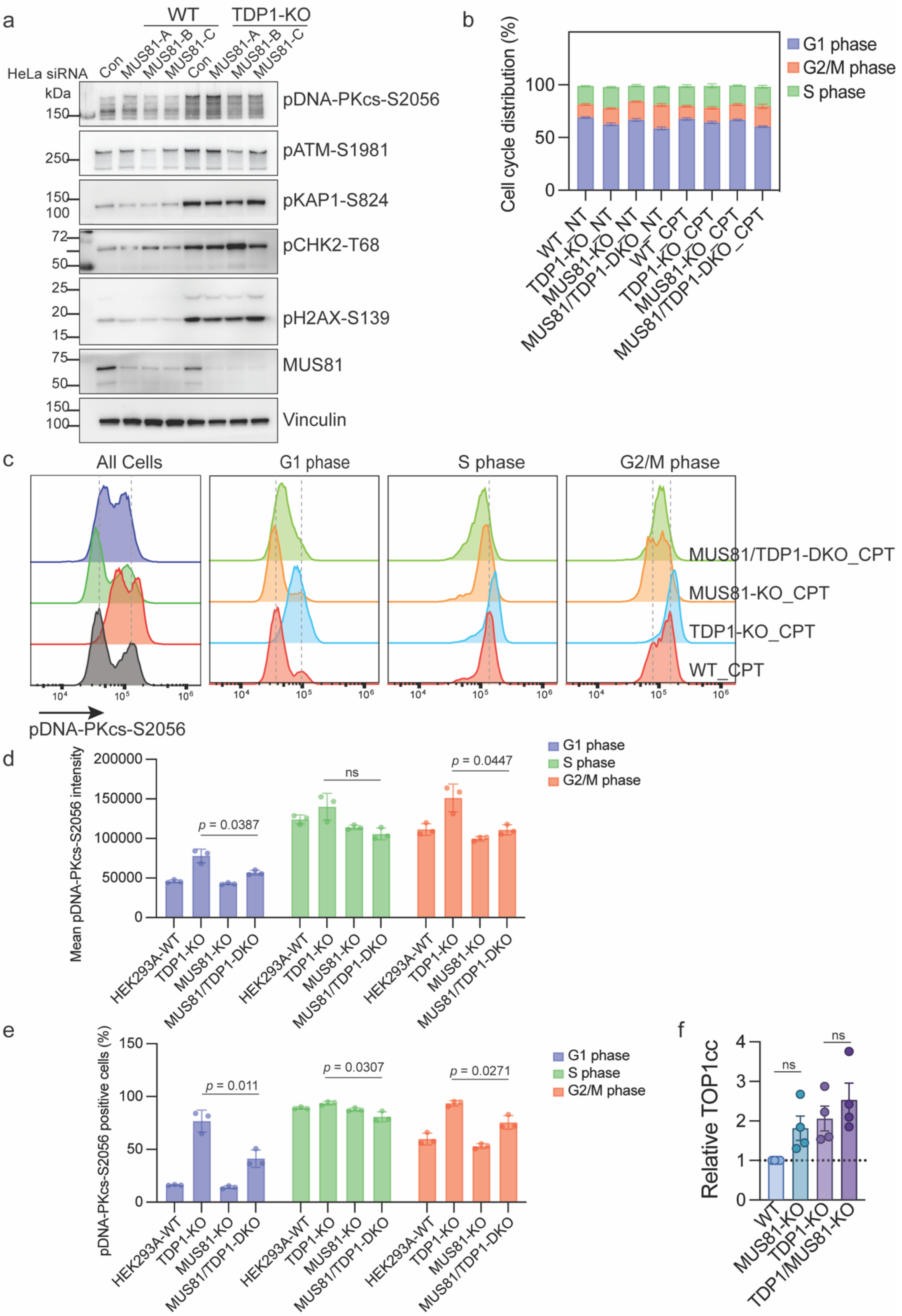

Supplementary Fig. 8: MUS81 caused a decreased pDNA-PKcs-S2056 signal in all cell cycle phases. a Control siRNA or siRNA against MUS81 (A, B, and C) were transfected into HeLa WT or HeLa TDP1-KO cells. 72 h after siRNA transfection, cells were treated with 10 μM CPT for 1 h. Whole-cell extracts were prepared and subjected to Western blotting with the indicated antibodies. Experiments were repeated at least three times, and similar results were obtained. **b** Cell cycle distribution of the flow cytometry analysis of WT, TDP1-KO, MUS81-KO, and MUS81/TDP1-DKO cells either not treated (NT) or treated with 10 μM CPT for 1 h. Data are presented as the mean ± SD (n = 3 biologically independent experiments). **c** Flow cytometry analysis of pDNA-PKcs-S2056 intensity in WT, TDP1-KO, MUS81-KO, and MUS81/TDP1-DKO cells treated with 10 μM CPT for 1 h. Cells from G1, G2/M, or S phase were independently presented. **d** Quantification of **c**. Mean pDNA-PKcs-S2056 intensity from three independent experiments is shown in a bar chart (mean ± SD, n = 3 biologically independent experiments). A two-tailed unpaired *t* test with Welch's correction was used for statistical analysis. ns, not significant. **e** Percentage of pDNA-PKcs-S2056-positive cells from three independent experiments was shown in a bar chart (mean ± SD, n = 3 biologically independent experiments). A two-tailed unpaired *t* test with Welch's correction was used for statistical analysis. **f** A RADAR assay was performed after treating the indicated cells with 10 μM CPT for 1 h to assess TOP1cc accumulation. Data are represented as mean ± SEM (n = 4 biologically independent experiments). A two-tailed unpaired *t* test with Welch's correction was used for statistical analysis. ns, not significant.
