# Supplementary material for: TDP1-independent pathways in the process and repair of TOP1-induced DNA damage
Source: Nat Commun. 2022 Jul 22;13:4240. doi: 10.1038/s41467-022-31801-7 (PMC9307636; doi:10.1038/s41467-022-31801-7)
Supplement: Supplementary file 2 — Description of Additional Supplementary Files [file 41467_2022_31801_MOESM2_ESM.pdf]

Description of Additional Supplementary Files

File Name: Supplementary Data 1
Description: Raw sgRNA counts in the CRISPR screens.

File Name: Supplementary Data 2
Description: Normalized sgRNA counts in the CRISPR screens.

File Name: Supplementary Data 3
Description: Drug Z score ranking of CPT co-essential genes in HEK293A-WT cells.

File Name: Supplementary Data 4
Description: Drug Z score ranking of CPT co-essential genes in TDP1-KO cells.

File Name: Supplementary Data 5
Description: Drug Z score ranking of TDP1 co-essential genes.

File Name: Supplementary Data 6
Description: Drug Z score ranking of TDP1 and CPT combined co-essential genes.

File Name: Supplementary Data 7
Description: KEGG pathway analysis of genes whose loss of function led to CPT sensitivity ($p < 0.01$) in WT and TDP1-KO cells.
