# Supplementary material for: TDP1-independent pathways in the process and repair of TOP1-induced DNA damage
Source: Nat Commun. 2022 Jul 22;13:4240. doi: 10.1038/s41467-022-31801-7 (PMC9307636; doi:10.1038/s41467-022-31801-7)
Supplement: Supplementary file 10 — Reporting Summary [file 41467_2022_31801_MOESM10_ESM.pdf]

nature portfolio

Corresponding author(s): Junjie Chen
Last updated by author(s): Jun 22, 2022

Reporting Summary

Nature Portfolio wishes to improve the reproducibility of the work that we publish. This form provides structure for consistency and transparency in reporting. For further information on Nature Portfolio policies, see our Editorial Policies and the Editorial Policy Checklist.

Statistics

For all statistical analyses, confirm that the following items are present in the figure legend, table legend, main text, or Methods section.

n/a	Confirmed	
☐	☒	The exact sample size (*n*) for each experimental group/condition, given as a discrete number and unit of measurement
☐	☒	A statement on whether measurements were taken from distinct samples or whether the same sample was measured repeatedly
☐	☒	The statistical test(s) used AND whether they are one- or two-sided<br>*Only common tests should be described solely by name; describe more complex techniques in the Methods section.*
☒	☐	A description of all covariates tested
☐	☒	A description of any assumptions or corrections, such as tests of normality and adjustment for multiple comparisons
☐	☒	A full description of the statistical parameters including central tendency (e.g. means) or other basic estimates (e.g. regression coefficient) AND variation (e.g. standard deviation) or associated estimates of uncertainty (e.g. confidence intervals)
☐	☒	For null hypothesis testing, the test statistic (e.g. *F*, *t*, *r*) with confidence intervals, effect sizes, degrees of freedom and *P* value noted<br>*Give P values as exact values whenever suitable.*
☒	☐	For Bayesian analysis, information on the choice of priors and Markov chain Monte Carlo settings
☒	☐	For hierarchical and complex designs, identification of the appropriate level for tests and full reporting of outcomes
☒	☐	Estimates of effect sizes (e.g. Cohen's *d*, Pearson's *r*), indicating how they were calculated

Our web collection on statistics for biologists contains articles on many of the points above.

Software and code

Policy information about availability of computer code

Data collection	CellTiter-Glo assay results were detected by a BioTek Synergy™ 2 Multi-Mode Microplate Reader. DNA concentration were determined by NanoDrop spectrophotometer (Thermo Scientific). Genome-wide CRISPR/Cas9 screens were sequenced by a Illumina HiSeq 2500 system. Comet assay images were captured by Nikon 90i microscope. FACS were conducted on a BD C6 flow cytometer (Becton Dickinson) or Attune Flow cytometers (ThermoFisher).
Data analysis	Statistical analysis was performed using GraphPad Prism 8.0.0. FACS data analysis was performed with FlowJo 10.6.1. Comet assay images were analyzed with OpenComet (v1.3.1 from https://cometbio.org). Quantification of the slot blot results was analyzed with Image J software (version 1.50i). KEGG pathway analysis was performed with DAVID Bioinformatics Resources 6.8, NIAID/NIH (https://david.ncifcrf.gov/summary.jsp). Genome-wide CRISPR/Cas9 screens results were analyzed with MAGeCK (https://sourceforge.net/p/mageck/wiki/Home/) and drug Z (https://github.com/hart-lab/drugz).

For manuscripts utilizing custom algorithms or software that are central to the research but not yet described in published literature, software must be made available to editors and reviewers. We strongly encourage code deposition in a community repository (e.g. GitHub). See the Nature Portfolio guidelines for submitting code & software for further information.

Data

Policy information about availability of data

All manuscripts must include a data availability statement. This statement should provide the following information, where applicable:

- Accession codes, unique identifiers, or web links for publicly available datasets
- A description of any restrictions on data availability
- For clinical datasets or third party data, please ensure that the statement adheres to our policy

The genome-wide CRISPR/Cas9 screen data generated in this study are provided in the Supplementary data files. Source data are provided with this paper.

Field-specific reporting

Please select the one below that is the best fit for your research. If you are not sure, read the appropriate sections before making your selection.

[x] Life sciences [ ] Behavioural & social sciences [ ] Ecological, evolutionary & environmental sciences

For a reference copy of the document with all sections, see nature.com/documents/nr-reporting-summary-flat.pdf

Life sciences study design

All studies must disclose on these points even when the disclosure is negative.

Sample size	No statistical methods was used to predetermine sample size. In most experiments, like Western Blot, Slot Blot, CRISPR screening, cell proliferation assay, FACS assay, and comet assay, at least three independent biological replicates were conducted, selected based on standard practice in the field. For comet assay, more than 200 cells were examined for each independent experiments for statistical analyses. Statistical analysis (as described in respective figure legends) was used to calculate statistical significance of obtained results. The individual p-values are indicated in figures or in figure legends.
Data exclusions	No data were excluded from the analyses.
Replication	All the experiments were repeated at least three times, and similar results were obtained from each independent experiments.
Randomization	For comet assay, images were randomly taken from left to right of the slide, and all images taken were used for statistical analysis. Randomization was not applicable to other studies as we only compare one factor in specific indicated cell lines at a time.
Blinding	Blinding was applied to quantification of slot blot results, CRISPR/Cas9 screens data analysis, and comet assay images capture and analysis. Blinding was not applied for other experiments as all data were obtained and presented in an unbiased way.

Reporting for specific materials, systems and methods

We require information from authors about some types of materials, experimental systems and methods used in many studies. Here, indicate whether each material, system or method listed is relevant to your study. If you are not sure if a list item applies to your research, read the appropriate section before selecting a response.

Materials & experimental systems

n/a	Involved in the study	
[ ]	[x]	Antibodies
[ ]	[x]	Eukaryotic cell lines
[x]	[ ]	Palaeontology and archaeology
[x]	[ ]	Animals and other organisms
[x]	[ ]	Human research participants
[x]	[ ]	Clinical data
[x]	[ ]	Dual use research of concern

Methods

n/a	Involved in the study	
[x]	[ ]	ChIP-seq
[ ]	[x]	Flow cytometry
[x]	[ ]	MRI-based neuroimaging

Antibodies

Antibodies used	Primary antibodies used for western blot:<br>TOP1cc (Clone 1.1A, MABE1084; Millipore; 1: 1000 dilution),<br>dsDNA (ab27156; Abcam; 1: 5000 dilution),<br>phospho-DNA-PKcs (S2056, ab18192; Abcam; 1: 1000 dilution),<br>phospho-KAP1 (S824, 4127S; Cell Signaling Technology; 1: 2000 dilution),<br>phospho-ATM (S1981, ab81292; Abcam; 1: 1000 dilution),

phospho-ATM (S1981, 13050S; Cell Signaling Technology; 1: 1000 dilution),
phospho-Chk2 (Thr68, 2661S; Cell Signaling Technology; 1: 1000 dilution),
phospho-H2AX (S139, 9718S; Cell Signaling Technology; 1: 1000 dilution),
phospho-Chk1 (Ser345, 2348S; Cell Signaling Technology; 1: 1000 dilution),
phospho-Chk1 (Ser317, 12302S; Cell Signaling Technology; 1: 1000 dilution),
phospho-RPA32 (S4/S8, A300-245A; Bethyl Laboratories; 1: 1000 dilution),
Chk2 (6334S; Cell Signaling Technology; 1: 1000 dilution),
KAP1 (A300-274A; Bethyl Laboratories; 1: 1000 dilution),
TDP1 (sc-365674; Santa Cruz Biotechnology; 1: 500 dilution),
TDP2 (sc-377280; Santa Cruz Biotechnology; 1: 500 dilution),
APEX1 (4128S; Cell Signaling Technology; 1: 1000 dilution),
XPF (A301-315A; Bethyl Laboratories; 1: 1000 dilution),
MUS81 (sc-53382; Santa Cruz Biotechnology; 1: 500 dilution),
SLX4 (in-house developed antibody against the antigen comprising SLX4231-460; 1: 1000 dilution),
EME1 (ab88878; Abcam; 1: 1000 dilution),
EME2 (NBP3-04534-100UL; Novus Biologicals; 1: 1000 dilution),
MRE11 (NB100-142; Novus Biologicals; 1: 1000 dilution),
CtIP (9201S; Cell Signaling Technology; 1: 1000 dilution),
DNA2 (ab96488, Abcam; 1: 1000 dilution),
H3 (ab1791; Abcam; 1: 2000 dilution),
Actin (A5441-100UL; Sigma-Aldrich; 1: 4000 dilution),
Vinculin (V9131; Sigma-Aldrich; 1: 4000 dilution),
Tubulin (T6199-200UL; Sigma-Aldrich; 1: 4000 dilution),

Primary and secondary antibodies used for FACS:
phospho-DNA-PKcs (S2056, ab18192; Abcam; 1: 1000 dilution),
phospho-H2AX (S139, 05-636l; Millipore; 1: 1000 dilution),
goat anti-rabbit IgG (H+L) cross-adsorbed secondary antibody, FITC (F-2765; Thermo Fisher Scientific; 1: 1000 dilution),
goat anti-mouse IgG (H+L) highly cross-adsorbed secondary antibody, and Alexa Fluor™ Plus 488 (A32723, Thermo Fisher Scientific; 1: 1000 dilution).

Validation

All commercial antibodies were validated by suppliers and/or prior references. In house using of the antibodies were validated further by inhibitor treatment, siRNA or knockout studies.
TOP1cc (Clone 1.1A, MABE1084; Millipore). https://www.emdmillipore.com/US/en/product/Anti-Topoisomerase-I-DNA-Covalent-Complexes-Antibody-clone-1.1A,MM_NF-MABE1084?ReferrerURL=https%3A%2F%2Fwww.google.com%2F&bd=1. Ref: Patel, A.G. et al. Immunodetection of human topoisomerase I-DNA covalent complexes. Nucleic Acids Res 44, 2816-2826 (2016).
dsDNA (ab27156; Abcam). https://www.abcam.com/ds-dna-antibody-35i9-dna-bsa-and-azide-free-ab27156.html.
phospho-DNA-PKcs (S2056, ab18192; Abcam). https://www.abcam.com/dna-pkcs-phospho-s2056-antibody-ab18192.html. The specificity of phospho-DNA-PKcs antibody was further confirmed in this study by Western Blot of DNAPK inhibitor treatment in Fig. 2a and Supplementary Fig. 2b.
phospho-KAP1 (S824, 4127S; Cell Signaling Technology). https://www.abcam.com/kap1-phospho-s824-antibody-ab70369.html.
phospho-ATM (S1981, ab81292; Abcam). https://www.abcam.com/atm-phospho-s1981-antibody-ep1890y-ab81292.html. The specificity of phospho-ATM antibody was further confirmed in this study by Western Blot of ATM inhibitor treatment in Fig. 2a and Supplementary Fig. 2b.
phospho-Chk2 (Thr68, 2661S; Cell Signaling Technology). https://www.cellsignal.com/products/primary-antibodies/phospho-chk2-thr68-antibody/2661.
phospho-H2AX (S139, 9718S; Cell Signaling Technology). https://www.cellsignal.com/products/primary-antibodies/phospho-histone-h2a-x-ser139-20e3-rabbit-mab/9718.
phospho-Chk1 (Ser345, 2348S; Cell Signaling Technology). https://www.cellsignal.com/products/primary-antibodies/phospho-chk1-ser345-133d3-rabbit-mab/2348.
phospho-Chk1 (Ser317, 12302S; Cell Signaling Technology). https://www.cellsignal.com/products/primary-antibodies/phospho-chk1-ser317-d12h3-xp-rabbit-mab/12302.
phospho-RPA32 (S4/S8, A300-245A; Bethyl Laboratories). https://www.thermofisher.com/antibody/product/Phospho-RPA32-Ser4-Ser8-Antibody-Polyclonal/A300-245A.
Chk2 (6334S; Cell Signaling Technology). https://www.cellsignal.com/products/primary-antibodies/chk2-d9c6-xp-rabbit-mab/6334.
KAP1 (A300-274A; Bethyl Laboratories). https://www.thermofisher.com/antibody/product/KAP-1-Antibody-Polyclonal/A300-274A.
TDP1 (sc-365674; Santa Cruz Biotechnology). https://www.scbt.com/p/tdp1-antibody-c-3;jsessionid=fHiCkK68YwwfpBvQTSH0C20_eLj6iZe6Ei361JxF2mA3T5adVKgy!-730452933. The specificity of TDP1 antibody was further confirmed in this study by Western Blot of knockout of TDP1 Supplementary Fig. 5a.
TDP2 (sc-377280; Santa Cruz Biotechnology). https://www.scbt.com/zh/p/tdp2-antibody-h-6. The specificity of TDP2 antibody was further confirmed in this study by Western Blot of knockout of TDP2 in Fig. 6f.
APEX1 (4128S; Cell Signaling Technology). https://www.cellsignal.com/products/primary-antibodies/ape1-antibody/4128. The specificity of APEX1 antibody was further confirmed in this study by Western Blot of knockout or knockdown of APEX1 in Fig. 5j, and Supplementary Fig. 5a.
XPF (A301-315A; Bethyl Laboratories). https://www.thermofisher.com/antibody/product/XPF-ERCC4-Antibody-Polyclonal/A301-315A. The specificity of XPF antibody was further confirmed in this study by Western Blot of knockout of XPF in Fig. 6a.
MUS81 (sc-53382; Santa Cruz Biotechnology). https://www.scbt.com/zh/p/mus81-antibody-mta30-2g10-3. The specificity of MUS81 antibody was further confirmed in this study by Western Blot of knockout or knockdown of MUS81 in Fig. 7a and Supplementary Fig.

8a.
SLX4 (in-house developed antibody against the antigen comprising SLX4231-460). Ref: Zhang, H. et al. SLX4IP acts with SLX4 and XPF-ERCC1 to promote interstrand crosslink repair. Nucleic Acids Res 47, 10181-10201 (2019). The specificity of SLX4 antibody was further confirmed in this study by Western Blot of knockdown of SLX4 in Fig. 6b.
EME1 (ab88878; Abcam). https://www.abcam.com/eme1-antibody-ab88878.html. The specificity of EME1 antibody was further confirmed in this study by Western Blot of knockdown of EME1 in Fig. 7b.
EME2 (NBP3-04534-100UL; Novus Biologicals). https://www.novusbio.com/products/eme2-antibody_nbp3-04534. The specificity of EME2 antibody was further confirmed in this study by Western Blot of knockdown of EME2 in Fig. 7c.
MRE11 (NB100-142; Novus Biologicals). https://www.novusbio.com/products/mre11-antibody_nb100-142. The specificity of MRE11 antibody was further confirmed in this study by Western Blot of knockdown of MRE11 in Fig. 6c.
CtIP (9201S; Cell Signaling Technology). https://www.cellsignal.com/products/primary-antibodies/ctip-d76f7-rabbit-mab/9201. The specificity of CtIP antibody was further confirmed in this study by Western Blot of knockdown of CtIP in Fig. 6d.
DNA2 (ab96488, Abcam). https://www.abcam.com/dna2-antibody-ab96488.html. The specificity of DNA2 antibody was further confirmed in this study by Western Blot of knockdown of DNA2 in Fig. 6e.
H3 (ab1791; Abcam). https://www.abcam.com/histone-h3-antibody-nuclear-marker-and-chip-grade-ab1791.html.
Actin (A5441-100UL; Sigma-Aldrich). https://www.sigmaaldrich.com/US/en/product/sigma/a5441.
Vinculin (V9131; Sigma-Aldrich). https://www.sigmaaldrich.com/US/en/product/sigma/v9131.
Tubulin (T6199-200UL; Sigma-Aldrich). https://www.sigmaaldrich.com/US/en/product/sigma/t6199.

Primary and secondary antibodies used for FACS:
phospho-DNA-PKcs (S2056, ab18192; Abcam). https://www.abcam.com/dna-pkcs-phospho-s2056-antibody-ab18192.html.
phospho-H2AX (S139, 05-636l). https://www.emdmillipore.com/US/en/product/Anti-phospho-Histone-H2A.X-Ser139-Antibody-clone-JBW301,MM_NF-05-636?ReferrerURL=https%3A%2F%2Fwww.google.com%2F&bd=1.
goat anti-rabbit IgG (H+L) cross-adsorbed secondary antibody, FITC (F-2765; Thermo Fisher Scientific). https://www.thermofisher.com/antibody/product/Goat-anti-Rabbit-IgG-H-L-Cross-Adsorbed-Secondary-Antibody-Polyclonal/F-2765.
goat anti-mouse IgG (H+L) highly cross-adsorbed secondary antibody, and Alexa Fluor™ Plus 488 (A32723, Thermo Fisher Scientific). https://www.thermofisher.com/antibody/product/Goat-anti-Mouse-IgG-H-L-Highly-Cross-Adsorbed-Secondary-Antibody-Polyclonal/A32723.

Eukaryotic cell lines

Policy information about cell lines

Cell line source(s)	HEK293A cells were purchased from ThermoFisher (R70507). HEK293T, and HeLa cells were purchased from the ATCC (Manassas, VA). XPF-KO and MUS81-KO cells were generated by Huimin Zhang previously in the lab as described in Zhang H, et al. Nucleic Acids Res 47, 10181-10201 (2019). Other knockout cell lines were generated in the lab by using CRISPR-Cas9 as described in the material and methods.
Authentication	HEK293T, and HeLa cell line were authenticated by STR profiling by ATCC. HEK293A cell line were authenticated by morphology by ThermoFisher.CRISPR-Cas9 based generation of knockout cell lines were validated by western blot and sequencing.
Mycoplasma contamination	Cell lines were all tested negative for mycoplasma using PCR based mycoplasma detection method.
Commonly misidentified lines (See ICLAC register)	No commonly misidentified cell lines were used in this study.

Flow Cytometry

Plots

Confirm that:

- [x] The axis labels state the marker and fluorochrome used (e.g. CD4-FITC).
- [x] The axis scales are clearly visible. Include numbers along axes only for bottom left plot of group (a 'group' is an analysis of identical markers).
- [x] All plots are contour plots with outliers or pseudocolor plots.
- [x] A numerical value for number of cells or percentage (with statistics) is provided.

Methodology

Sample preparation	Cells were pre-treated with indicated drugs as shown in each figure. After treatment, cells were collected and fixed with ice-cold 70% ethanol at 4°C overnight. Cells were then washed with phosphate-buffered saline (PBS), and permeabilized with 0.5% Triton X-100/PBS at ambient temperature for 10 min. After washed with PBS, cells were blocked with 4% bovine serum albumin (BSA)/PBS for 1 h, and incubated with phospho-DNA-PKcs (S2056, ab18192; Abcam) anitibody diluted in 4% BSA/PBS for 1 h at ambient temperature. Cells were washed with PBS for three times, and incubated with fluorescently labeled secondary antibodies diluted in 4% BSA/PBS for 1 h. Cells were stained with propidium iodide (PI; 20 µg/ml) and RNase A (10 µg/ml) before analysis. For two-color competitive growth assay, live cells were analyzed directly by Attune Flow cytometers (ThermoFisher).

Instrument	BD C6 flow cytometer (Becton Dickinson) or Attune Flow cytometers (ThermoFisher).
Software	FlowJo 10.6.1
Cell population abundance	At least 10,000 events were recorded for each sample.
Gating strategy	Cells were first gated for live cells using FSC-A/SSC-A, followed by gating for single cells (FSC-A/FSC-H). The PI histogram was used to gate %G1, %S, %G2/M phase cell populations.

☒ Tick this box to confirm that a figure exemplifying the gating strategy is provided in the Supplementary Information.
